# Protective effect of Huashi Baidu formula against AKI and active ingredients that target SphK1 and PAI-1

**DOI:** 10.1186/s13020-024-01024-7

**Published:** 2024-11-01

**Authors:** Yute Zhong, Xia Du, Ping Wang, Weijie Li, Cong Xia, Dan Wu, Hong Jiang, Haiyu Xu, Luqi Huang

**Affiliations:** 1 College of Chinese Medicinal Materials, Jilin Agricultural University, Changchun, 130118 Jilin China; 2 Institute of Chinese Materia Medica, China Academy of Chinese Medical Sciences, Beijing, 100700 China; 3 Institute of Traditional Chinese Medicine, Shaanxi Academy of Traditional Chinese Medicine, Xi’an, China; 4 State Key Laboratory for Quality Ensurance and Sustainable Use of Dao-Di Herbs, Institute of Chinese Materia Medica, China Academy of Chinese Medical Sciences, Beijing, 100700 China; 5 National Resource Center for Chinese Materia Medica, China Academy of Chinese Medical Sciences, Beijing, 100700 China

**Keywords:** Huashi Baidu, Acute kidney injury, Molecular mechanism, Active ingredients

## Abstract

**Background:**

Huashi Baidu Formula (HBF) is a clinical formula known for its efficacy against coronavirus disease 2019 (COVID-19). HBF may reduce the number of patients with abnormal serum creatinine while improving respiratory symptoms, suggesting that this formula may have potential for treating acute kidney injury (AKI). However, the protective effect of HBF on AKI has not been definitively confirmed, and the mechanism remains unclear. Therefore, the present study explored the renoprotective effects and molecular mechanisms of HBF and screened for its active ingredients to identify new potential applications of renoprotection by HBF.

**Methods:**

The present study first assessed the protective effects of HBF on AKI in a DOX-induced mouse model. Then, RNA-seq and bioinformatics analyses were used to explore the related pathological processes and potential molecular mechanisms, which were subsequently validated using qRT-PCR and Western blotting. Furthermore, candidate compounds with potential binding affinity to two pivotal targets, sphingosine kinase 1 (SphK1) and plasminogen activator inhibitor-1 (PAI-1), were screened from the 29 constituents present in the blood using Microscale Thermophoresis (MST). Finally, to identify the active ingredients, the candidate components were re-screened using the SphK1 kinase activity detection system or the uPA/PAI-1 substrate colorimetric assay system.

**Results:**

In the DOX-induced AKI mouse model, therapeutic administration of HBF significantly reduced the levels of CRE, BUN, TNF-α, IL-1β, IL-6, and UA in plasma and the levels of MDA, T-CHO, and TG in kidney tissue. Additionally, the levels of TP and Alb in plasma and SOD and CAT in the kidney tissue were significantly increased. Histopathological assessment revealed that HBF reduced tubular vacuolation, renal interstitial inflammatory cell infiltration, tubular atrophy, and positive staining of renal interstitial collagen. RNA-seq and bioinformatics analyses showed that oxidative stress, the immune-inflammatory response, and extracellular matrix (ECM) formation could be the pathological processes that HBF targets to exerts its renoprotective effects. Furthermore, HBF regulated the APJ/SPHK1/NF-κB and APJ/PAI-1/TGFβ signaling axes and reduced the phosphorylation levels of NF-κB p65 and SMAD2 and the expression of cytokines and the ECM downstream of the axis. Finally, six SphK1 inhibitors (paeoniflorin, astragalin, emodin, glycyrrhisoflavone, quercetin, and liquiritigenin) and three PAI-1 inhibitors (glycyrrhisoflavone, licochalcone B, and isoliquiritigenin) were identified as potentially active ingredients in HBF.

**Conclusion:**

In brief, our investigation underscores the renoprotective effect of HBF in a DOX-induced AKI model mice, elucidating its mechanisms through distinct pathological processes and identifying key bioactive compounds. These findings offer new insights for broadening the clinical applications of HBF and unravelling its molecular mode of action.

**Supplementary Information:**

The online version contains supplementary material available at 10.1186/s13020-024-01024-7.

## Background

AKI is characterized by a rapid decline in renal function and is induced by factors such as sepsis, nephrotoxic drug effects, renal ischaemia/reperfusion, and cardiogenic shock [1, 2]. AKI surpasses breast cancer and heart failure in terms of mortality rates worldwide, persisting above 23% for the past five decades, with a prevalence of 30–60% in intensive care units (ICUs) [3–6]. Patients with persistent renal dysfunction for 3–7 days have a significantly reduced 1-year survival rate post-AKI. Survivors often face irreversible glomerular loss, renal fibrosis, compromised immunity, and increased susceptibility to chronic kidney failure, cardiovascular disease, stroke, and secondary infections [3, 7–9]. Current strategies for managing AKI primarily focus on addressing the underlying causes, including controlling blood pressure in cardiogenic AKI, providing anti-infective treatment in septic AKI, and discontinuing nephrotoxic medications in drug-induced AKI [3]. However, these approaches lack specificity and rely heavily on timely diagnosis, potentially missing the crucial intervention window [10]. And specific therapeutics for AKI prevention or treatment are currently unavailable.

Delving deeper into the pathophysiology of AKI, it becomes evident that the condition is governed by a quartet of critical processes: oxidative stress, inflammation, cellular death, and aberrant ECM formation, collectively orchestrating the renal dysfunction characteristic of this acute syndrome [11]. Oxidative stress, driven by the overproduction of reactive oxygen species (ROS), leads to cellular structural and functional damage by directly impairing cell membranes, proteins, and DNA, resulting in cellular dysfunction [12]. Inflammatory responses in AKI involve the release of cytokines which increase vascular permeability, causing tissue edema and infiltration of inflammatory cells [13]. Cell death is another critical mechanism of tissue injury in AKI, activated through various signaling pathways leading to the loss of renal tubular epithelial cells. The formation of ECM plays a crucial role in kidney repair following AKI, but abnormal ECM deposition can lead to renal fibrosis and structural changes, further impairing renal function. These interconnected processes collectively drive the development and progression of AKI. Therefore, targeting signaling pathways related to these mechanisms with therapeutic agents may be a promising approach for AKI prevention and management [14].

Ancient Chinese medical texts, such as *Huangdi’s Internal Classic* (Huang Di Nei Jing), associate AKI symptoms with “dribbling and retention of urine”, viewing AKI in traditional Chinese medicine (TCM) as a dampness-heat syndrome. Treatment focuses on heat clearance, dampness removal, diuresis promotion, and blood cooling, with various effective formulas following this approach [15, 16]. Recent research highlights TCM’s advantages in managing AKI [17]. HBF is a clinically effective formula for treating COVID-19 [18–24] and comprises 14 herbs, including *Ephedrae Herba* (Mahuang, MH), *Pogostemonis Herba* (Guanghuoxiang, GHX), *Gypsum Fibrosum* (Shigao, SG), *Armeniacae Semen Amarum* (Kuxingren, KXR), *Pinelliae Rhizoma* (Banxia, BX), *Magnoliae Officinalis Cortex* (Houpo, HP), *Atractylodis Rhizoma* (Cangzhu, CZ), *Tsaoko Fructus* (Caoguo, CG), *Poria* (Fuling, FL), *Astragali Radix* (Huangqi, HQ), *Descurainiae Semen* (Nantinglizi, TLZ), *Paeoniae Radix Rubra* (Chishao, CS), *Rhei Radix et Rhizoma* (Dahuang, DH), and *Glycyrrhizae Radix et Rhizoma* (Gancao, GC). Research on the syndrome typing of COVID-19 indicate that it can be categorized under the dampness-heat syndrome [25, 26]. Actually, among the several herbs compromised HBF, such as Mahuang and Guanghuoxiang, are known to affect dampness and heat syndromes. Interestingly, patients receiving lopinavir-ritonavir treatment show increased blood Cre abnormalities, a trend not observed with concurrent HBF treatment, suggesting potential renal safety enhancement when HBF is combined with COVID-19 therapy [19, 27]. Furthermore, eight of the 14 herbs in HBF, including MH [28], CZ [29], CG [30], FL [31], HQ [32], CS [33], DH [34], and GC [35], have documented effects on renal function injury, suggesting a potential protective effect of HBF on renal function.

However, the renoprotective efficacy of HBF remains to be verified. This study aimed to validate the renoprotective capabilities of HBF in AKI using animal models, to uncover and verify its molecular mechanism, and to identify the active ingredients linked to this mechanism. These efforts may expand the scope of application of the formula and establish a foundation for exploring its efficacy and molecular mechanism.

## Materials and methods

### Animal experiments

Male C57BL/6 mice (8–10 weeks old) were obtained from Guangzhou Viton Lihua Laboratory Animal Technology Co. Ltd. and housed under specific pathogen-free (SPF) conditions. The animal experiments were approved by the Animal Experimental Ethics Committee of Guangzhou Huateng Biomedical Technology Co. Ltd. (approval number: HTSW230103, January 3, 2023). The mice were acclimated in a quarantine room for 1 week before the experiment, with free access to water and pellet food.

To evaluate the protective effects of HBF on kidney function, a drug-induced mouse model of AKI was created using DOX. The mice (n = 6 each) were assigned to one of six groups: Ctrl, DOX, low-dose HBF (HBF-L), medium-dose HBF (HBF-M), high-dose HBF (HBF-H), or prednisone (PRS). Except for the Ctrl group, all the mice received intraperitoneal injections of 6 mg/kg DOX on Days 0, 2, and 4 of the experiment to induce AKI. Treatments with HBF and PRS began on Day 1 and continued until the end of the study (Day 8). The doses of HBF administered to mice were determined on the basis of clinical human doses, with the medium dose considered equivalent to the clinical dose. The treatment doses for the HBF groups were set at 1.3 g/kg, 2.6 g/kg, and 5.2 g/kg. The equivalent dose of PRS, which is based on human dosing, was calculated as 4.55 mg/kg. After the experiment, the animals were humanely euthanized, and plasma and kidney tissue samples were collected for further analysis.

### Biomarker assessment

Renal function was evaluated by measuring the plasma levels of Cre and BUN. Hypoproteinaemia was determined by analysing plasma total protein (TP) and albumin (Alb) levels. The inflammatory response was assessed by evaluating interleukin-6 (IL-6), interleukin-1 beta (IL-1β), and tumour necrosis factor (TNF-α) levels. Oxidative stress injury was assessed by measuring MDA, SOD, and CAT in renal tissues using commercial kits. Kits for Cre, BUN, SOD, and CAT were purchased from Nanjing Jiancheng, while TP was analysed using a BCA assay kit purchased from Beyotime. ELISA kits for Alb, IL-6, IL-1β, and TNF-α were purchased from MEIMIAN, and were used following the manufacturer’s instructions.

For kidney tissue biomarker detection, prechilled saline was added at a ratio of 1:9 for homogenization. A 10% tissue homogenate was prepared using a tissue homogenizer at a low temperature. The homogenate was subjected to two rounds of centrifugation at 3,500 rpm for 10 min each at 4 °C. The resulting supernatant was then collected for testing.

### Histological analysis

Kidney tissues from the mice were fixed in 4% paraformaldehyde for 24 h, dehydrated, embedded in paraffin, and sectioned at a thickness of 4 mm. After deparaffinization, the sections were stained with H&E, PAS, and Masson staining kits and then examined under a microscope.

### RNA-Seq

Gene Denovo Biotechnology Co., Ltd. performed the RNA-seq analysis of mouse kidney tissue. The procedure involved extracting total RNA from mouse kidney tissue using the TRIzol method, quality-checking the sample, constructing libraries, sequencing RNA on the Illumina platform, representing gene expression in each sample by read count, and subsequently performing sequencing depth and transcript length corrections to obtain standardized FPKM values.

### Bioinformatic analysis

Sample relationships were analysed using principal component analysis (PCA) and differential gene analysis (DEGs) of the RNA-Seq results. DEGs were identified based on a significance threshold of P < 0.05. Genes displaying opposite expression patterns in different comparisons were categorized as reverse-regulated genes. Biological process (BP) terms relevant to oxidative stress, the immune response, the inflammatory response, cell death and survival, and ECM formation were obtained from the Gene Ontology (GO) database (https://www.geneontology.org/) to create the gene sets for pathological processes, which were presented in a Gene Matrix Transposed (GMT) file format for further gene set variation analysis (GSVA). The GSVA scores were used to measure the enrichment level of each pathological process across the samples, indicating the activation status of different pathological processes. Protein‒protein interaction (PPI) analysis was conducted on each pathological process gene set using STRING (https://cn.string-db.org/), and topological eigenvalues were calculated with the CytoHubba plug-in of CytoScape v3.9.1 software. Signal transduction pathway genes were collected from the Kyoto Encyclopedia of Genes and Genomes (KEGG) database (https://www.genome.jp/kegg/), GMT files of signal transduction pathways were created, and the enrichment level of signal transduction pathways associated with each pathology process by reverse-regulated genes was analysed via single-sample gene set enrichment analysis (ssGSEA) scores. Pathway crosstalk and regulatory direction were examined using the PathExNET online platform (https://pathexnet.cing-big.hpcf.cyi.ac.cy/#step-1). Pathways were considered to be relatively overexpressed when the combined FC > 1.0 and relatively underexpressed when the combined FC < 1.0.

### Quantitative real-time PCR assay

Total RNA was extracted from mouse kidney tissue samples (15–25 mg) via TRIzol reagent (DP424, TIANGEN, China), and RNA purity and concentration were assessed using a Nanodrop 2000 (Thermo, USA). The mRNA was converted to cDNA using FastKing gDNA Dispelling RT SuperMix (KR118-02; TIANGEN, China) for qPCR analysis. qPCR was conducted using a ChamQ SYBR qPCR Master Mix (Low ROX Premixed) kit (Q331-02, Vazyme, China) with the primer sequences detailed in Table S3.

### Immunofluorescence staining

As described in Sect. 2.3, mouse kidney tissues were fixed, dehydrated, paraffin-embedded, and sectioned. The tissue antigens were retrieved by microwave treatment with sodium citrate and then blocked with goat serum. This was followed by sequential steps: primary antibody incubation, washing, secondary antibody incubation, DAPI staining, and final observation of the staining using fluorescence microscopy.

### Western blotting

Mouse kidney tissues were lysed with RIPA buffer (P0013B, Beyotime, China) and homogenized with a tissue grinder, followed by a 30 min low-temperature incubation. The resulting lysate was centrifuged at 15,000 × g at 4 °C to collect the total protein extract. The total protein concentration was determined using a BCA protein assay kit (P0010, Beyotime, China). The samples were then standardized with PBS and loading buffer, separated on 8–12% SDS-PAGE gels, and transferred to PVDF membranes (ISEQ00010, Millipore, USA). After blocking with 5% skim milk, the membranes were incubated overnight at 4 °C with the following primary antibodies: p65 (80979–1-RR, Proteintech, China), p-p65 (bs-0982R, Bioss, China), IκB-α (10268–1-AP, Proteintech, China), p-IκB-α (2859, CST, USA), SMAD2/3 (sc-133098, Santa Cruz, USA), p-SMAD2 (18338, Santa Cruz, USA), PAI-1 (66261–1-Ig, Proteintech, China), and transforming growth factor beta-1 proprotein (TGF-β1) (A2124, ABclonal, China). GAPDH (bs-2188R, Bioss, China) was used as an internal control. Following three washes with TBST, the membranes were incubated with an HRP-conjugated secondary antibody at room temperature for 1.5 h. Finally, membrane development was conducted using an enhanced chemiluminescence (ECL) solution (A38554, Thermo, USA).

### Cell transfection and lysate preparation

HEK293T cells were cultured in Dulbecco’s modified Eagle’s medium (DMEM) supplemented with 10% foetal bovine serum and 1% penicillin‒streptomycin at 37 °C with 5% CO_2_. The cells were trypsinized with 0.05% trypsin–EDTA for passaging when they exceeded 80% confluency. One day before transfection, the cells were seeded in 6-well plates at a density of 6 × 10^5^ cells per well, and the transfections were performed when the cells reached 70–80% confluency in DMEM supplemented with 10% FBS and no antibiotics. The overexpression plasmids pCMV3-SPHK1-GFPSpark, pCMV3-SERPINE1-GFPSpark, pCMV3-C-GFPSpark, and pCMV3-SPHK1 and the control plasmid pCMV3 were purchased from Sino Biological Co. Lipofectamine 6000 was used as the transfection reagent.

The cells, whether they were transfected with plasmids or not, were initially detached from the culture medium. They were then washed with prechilled PBS buffer and centrifuged at 1200 × g for 5 min at 4 °C to harvest the cell pellet. The cells were subsequently lysed with RIPA buffer for 30–40 min in an ice bath, followed by centrifugation at 15,000 × g for 15 min at 4 °C. The resulting supernatant was subjected to protein concentration determination using a BCA protein assay kit and was then dispensed and frozen to generate the cell lysate.

### Protein purification-free MST molecular interaction

To identify bioactive compounds with nephroprotective effects in HBF, we established a protein purification-free MST molecular interaction system to screen compounds potentially targeting SphK1 and PAI-1, comprising cell lysates and compounds formulated with an interaction buffer. In addition, a compound library comprising 28 molecules was assembled using 60 prototype blood-entry compounds as the basis for HBF, which served as the primary target for screening in this study. Specific details can be found in Table S4 [23].

The interaction buffer for SphK1 consisted of 10 mM PBS (pH 7.2–7.4) with 6 mg/mL BSA, whereas for PAI-1, it was 10 mM PBS (pH 7.2–7.4). The final concentration of small molecules in the intercalation system was 200 μM, while the content of DMSO was 2%. After preparation, the system was incubated at room temperature for 10 min. The fluorescence signals emitted from the green fluorescent protein (GFP) tag were subsequently evaluated using the binding check mode of a Monolith instrument (Nanotemper, Germany). A signal-to-noise ratio exceeding 5 confirmed the affinity potential of the ligands for the receptors (GFP-SphK1 and GFP-PAI-1).

### ATP-dependent kinase activity assay

SphK1 is an ATP-dependent kinase that catalyses the transfer of the phosphate group from ATP to the hydroxyl group of the substrate sphingosine (Sph), leading to the conversion of Sph to sphingosine 1-phosphate (S1P). Thus, an ATP-dependent kinase activity assay system can be established to determine kinase activity by measuring the remaining ATP in the system. This study utilized SphK1-overexpressing cell lysate as the crude SphK1 enzyme mixture. The experiments were performed in all-white 96-well plates, and the enzyme-catalysed reaction buffer was 20 mM Tris–HCl buffer (pH 7.4, containing 10% glycerol, phosphorus, 1 mM β-mercaptoethanol, 10 mM MgCl_2_, and a 1 × phosphorylphosphatase inhibitor mixture). During the assay, 1 μL of compound or DMSO was mixed with 19 μL of the enzyme mixture and incubated at room temperature for 30 min. Subsequently, 5 μL of ATP-Sph substrate working solution (50 μM) was added for a further 30-min incubation, followed by detection of the residual ATP using the Kinase-Lumi^™^ kinase activity assay kit (S0158, Beyotime, China) through a multimode microplate reader (Varioskan LUX, Thermo, USA). The kinase activity was then calculated, with DMSO serving as the control.

$${\text{Kinase activity }}\left( {\text{\% }} \right) = \frac{{ATP_{Control} - ATP_{test} }}{{ATP_{Control} }} \times 100\%$$

### PAI-1 activity assay

The urokinase-type plasminogen activator (uPA) plays a specific catalytic role in the hydrolysis process of the small molecule peptide S-2444, leading to the production of p-nitroaniline (pNA), which has distinctive absorption characteristics at 405 nm. PAI-1 is an endogenous inhibitor of uPA that inhibits the hydrolytic activity of uPA towards S-2444. Therefore, the activity of PAI-1 can be characterized by detecting the production of pNA at 405 nm.

The experiments were performed in 96-well plates with transparent bottoms, comprising test wells (Compound), test positive control wells (Compound-Ctrl), test background control wells (Compound-Background), positive control wells (POS-Ctrl), negative control wells (NEG-Ctrl), background control wells (Background), and PAI-1 positive inhibitor (PAI-039) test wells. The enzymatic reaction buffer was 0.05 M Tris–HCl buffer (pH 8.0).

First, 1 μL of the compound was blended with 29 μL of the PAI-1 working solution, followed by a 10-min incubation at room temperature. Subsequently, 10 μL of the uPA working solution was added, and the mixture was incubated at 37 °C for 5 min to ensure complete inhibition of uPA by PAI-1. Finally, the S-2444 substrate was added, and the absorbance at 405 nm was determined using a multimode microplate reader in kinetic mode. The inhibition of PAI-1 activity by the compounds was expressed as a percentage of the relative PAI-1 activity and was calculated using the following equation:

$$PAI - 1 \,activity_{DMSO} \left( \% \right)\, = \,\left[ {\frac{{\Delta \left( {OD_{NEG - Ctrl} - OD_{Background} } \right)}}{{\Delta \left( {OD_{POS - Ctrl} - OD_{Background} } \right)}}} \right] \times 100\%$$

$$PAI - 1 \,activity_{compound} \left( \% \right) = \left[ {\frac{{\Delta \left( {OD_{Compound} - OD_{Compound - Background} } \right)}}{{\Delta \left( {OD_{Compound - Ctrl} - OD_{Compound - Background} } \right)}}} \right] \times 100\%$$

$$Relative \,PAI - 1 \,activity \left( \% \right) = \frac{{PAI - 1 activity_{compound} }}{{PAI - 1 activity_{DMSO} }}$$

### Statistical analysis

The data were analysed using GraphPad Prism 8.0 and are presented as the means ± SEMs. Normality and homogeneity of variance were assessed with F tests, and between-group differences were evaluated using standard one-way ANOVA or appropriate nonparametric tests. Post hoc multiple comparisons were performed via Dunnett’s or Duckey’s correction depending on variance homogeneity. Statistical significance was set at *p* < 0.05. Graphs were generated with GraphPad Prism 8.3.0 and Adobe Illustrator CS5.

## Results

### HBF protects against DOX-induced AKI in mice

**Fig. 1:**
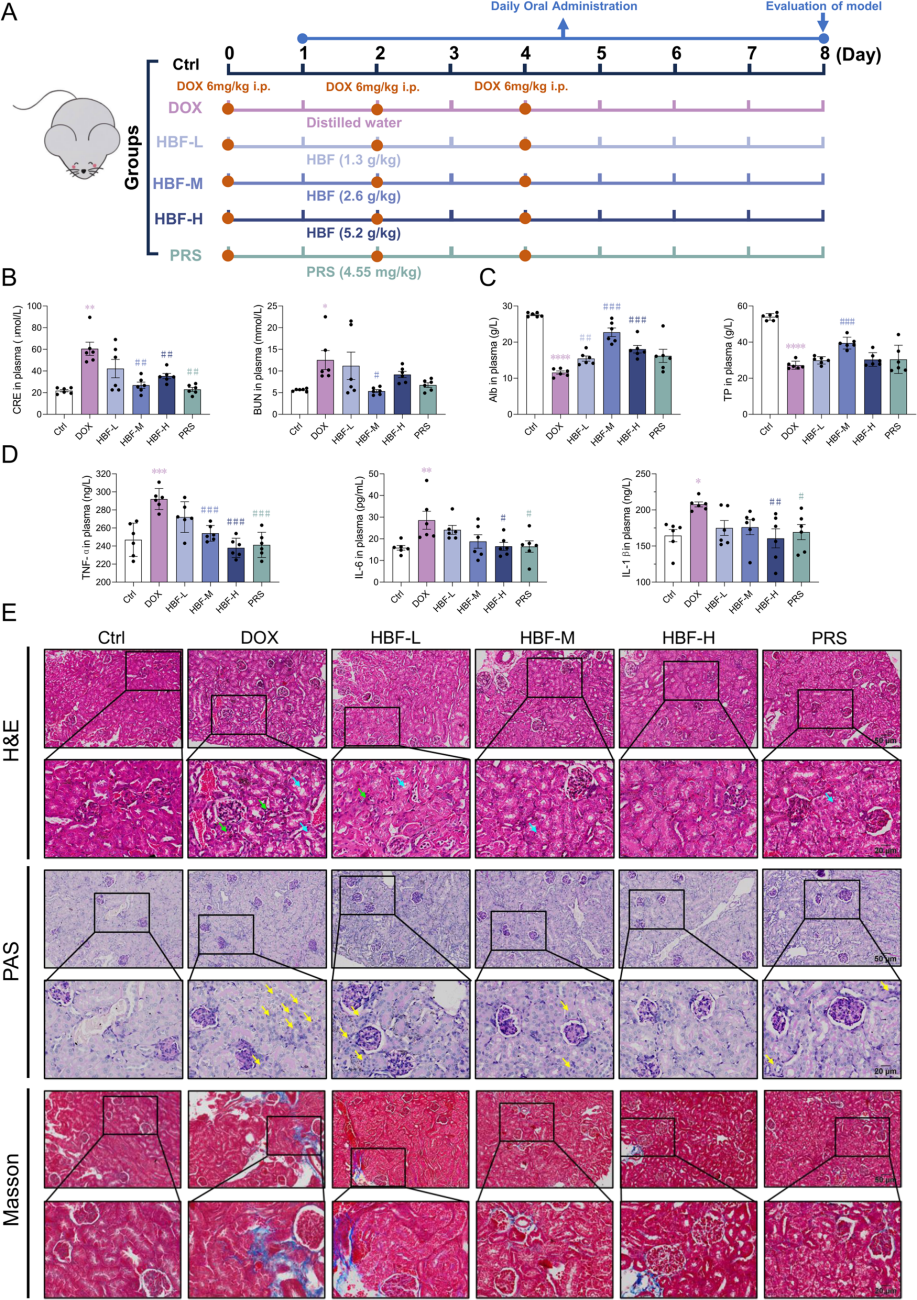
HBF administration protects against DOX-induced AKI in mice. **A** Schematic of the timing for inducing and administering treatments in the mouse AKI model. **B** Renal function indicators Cre and BUN in the mice in each group; n = 6 per group. **C** Hypoproteinaemia indicators TP and Alb in the plasma of the mice in each group, n = 6 per group. **D** Inflammatory factors in the plasma of each group; n = 6 per group. **E** H&E, PAS, and Masson staining of kidney sections from each group; n = 6 per group. (Data are presented as the mean ± SEM, *p < 0.05 vs. Ctrl; **p < 0.01 vs. Ctrl; ***p < 0.001 vs. Ctrl; ****p < 0.0001 vs. Ctrl, #*p* < 0.05 vs. DOX, ##*p* < 0.01 vs. DOX; ###p < 0.001 vs. DOX)

The renoprotective efficacy of HBF was assessed in a DOX-induced mouse model of AKI. The administration of the treatment started on the second day after the initial DOX induction and continued until the study’s completion (Fig. 1A). Elevated plasma Cre and BUN levels are markers of impaired renal function, and hypoproteinaemia is a relevant phenotype for the development of AKI. We observed a significant increase in the plasma Cre and BUN levels, as well as a notable reduction in the Alb and TP levels, in the DOX group, which were ameliorated by HBF and PRS administration, with the best recovery of the indices in the HBF-M group (Fig. 1B, C). The anti-inflammatory effect of HBF was also evaluated. The plasma inflammatory factor levels in the mice were significantly elevated in the DOX-induced model, indicating a systemic inflammatory response, which was ameliorated in the HBF-treated group (Fig. 1D).

H&E and PAS staining of renal tissue sections revealed that HBF alleviated DOX-induced pathological changes in mouse kidneys, including tubular vacuolar degeneration, inflammatory cell infiltration in the renal interstitium, and tubular atrophy. The Masson staining results demonstrated that HBF reduced interstitial collagen deposition (Fig. 1E). Tubular vacuolar degeneration is closely related to tubular epithelial cell death and oxidative stress injury, whereas inflammatory cell infiltration is related to the immune response and inflammatory response of the intrinsic cells of renal tissue. In summary, the various pathological processes involved in the renoprotective role of HBF include oxidative stress, immune and inflammatory responses, cell death and survival, and ECM formation.

### RNA-Seq data analysis

**Fig. 2:**
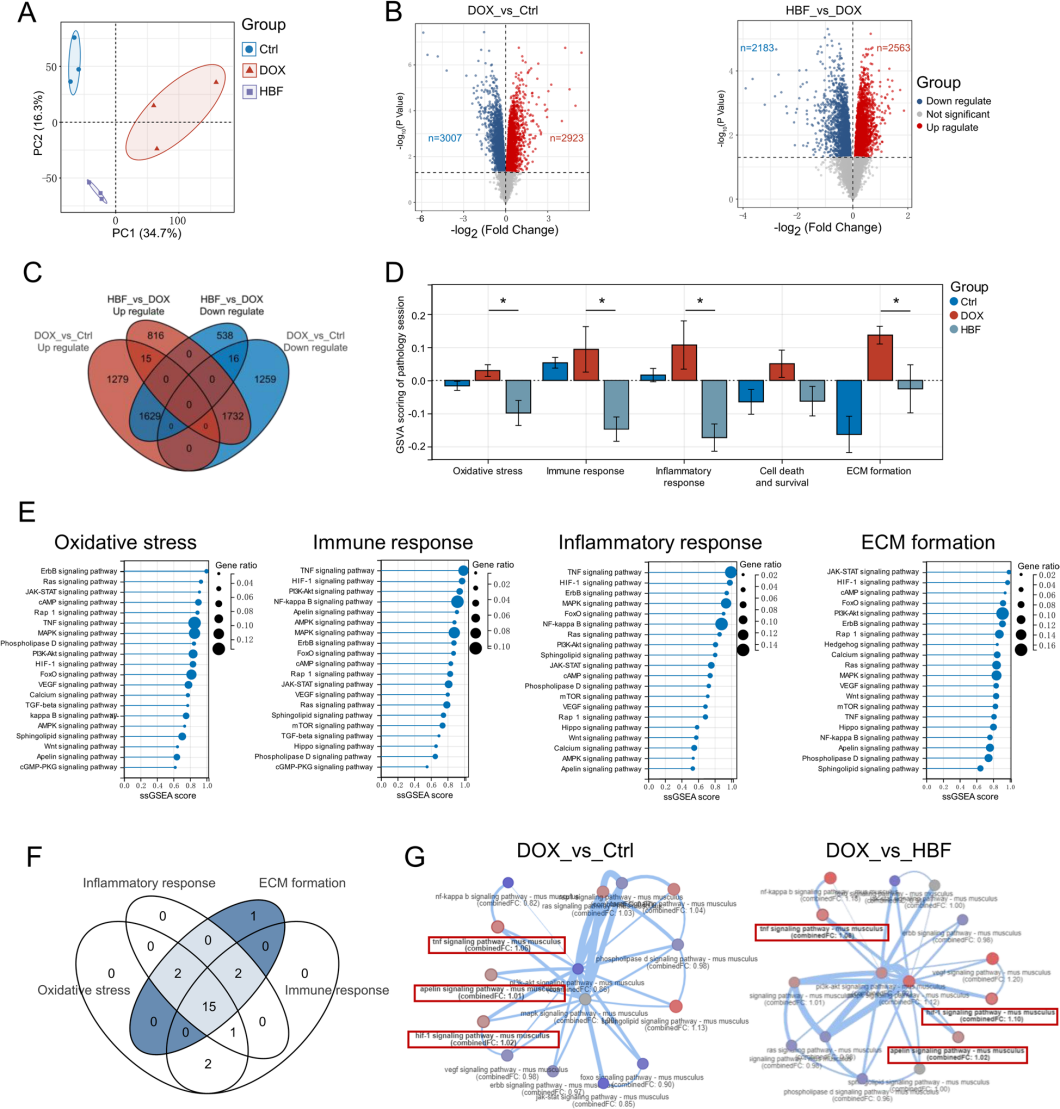
Analysis of RNA-Seq data for pathways related to HBF’s renoprotective effects. **A** PCA of the RNA-Seq data. **B** DEG analysis by RNA-Seq. **C** Venn diagram of DEGs. **D** ssGSEA scores for the signal transduction pathways of the reverse-regulated genes in each pathological process (data are presented as the means ± SD, *p < 0.05 vs. DOX). **E** Signaling transduction pathways in the top 20 ssGSEA scores in each pathology process. **F** Venn diagram of the top 20 signaling pathways associated with each pathological process. **G** PathExNET analysis of signal transduction pathways associated with the 4 pathological processes

To further explore the molecular mechanisms by which HBF exerts a protective effect on renal function, we conducted RNA-Seq and bioinformatics analysis on renal tissues obtained from experimental mice on the basis of the above studies. Preliminary PCA of the RNA-Seq data revealed distinct clustering of samples within the same experimental group and significant differences between various experimental groups in the principal component space (Fig. 2A).

The analysis of differentially expressed genes revealed that 2,923 genes were significantly upregulated (*p* < 0.05) and that 3,007 genes were significantly downregulated (*p* < 0.05) in the DOX group compared with the Ctrl group. Compared with those in the DOX group, 2,563 genes were significantly upregulated (*p* < 0.05), and 2,183 genes were significantly downregulated (*p* < 0.05) in the HBF group. Additionally, 3,361 genes exhibited reverse regulation in the HBF group. These findings demonstrate that HBF administration and DOX treatment significantly affected the gene expression pattern of mouse kidney tissue (Fig. 2B, C).

On the basis of prior research, we constructed gene sets specific to the pathological processes of interest using the GO database (Table S1). These tailored gene sets were subsequently utilized to conduct GSVA scoring on the RNA-Seq data. The findings revealed notable differences in the scores of oxidative stress, immune response, inflammatory response, and ECM formation between the HBF and DOX groups (Fig. 2D). These four pathological processes may be promising targets for treatment with HBF.

To investigate how HBF concurrently regulates these four pathological processes, we focused on signal transduction pathways known for their diverse biological function-regulating properties. PPI analysis was conducted on genes in each tailored gene set, and the reverse-regulated genes were extracted with their respective degree values, considering both gene expression differences and their pivotal positions in the pathways. Subsequently, signal transduction pathways were assessed using ssGSEA scoring, revealing that 15 out of the top 20 pathways were shared across all pathological processes (Fig. 2E, F). By analysing these 15 signaling pathways via PathExNET, we found that the apelin, TNF, and HIF-1α signaling pathways were enriched in pathway crosstalk and had combined FC values greater than 1 in the DOX vs. Ctrl and DOX vs. HBF groups, suggesting that the direction of regulation for these pathways was opposite that of the other two DOX groups (Fig. 2G and Table S2). Among them, we direct our attention to the apelin signaling pathway, as the pathway downstream of it was shown to be directly associated with renal disease in the KEGG pathway map. The pathway has been implicated as a pivotal mediator, with studies implicating its participation in renal oxidative stress, immune responses, inflammatory processes, and the etiology of ECM accumulation [36–38]. These biological mechanisms are highly relevant to the objectives of the current research endeavor.

**Fig. 3:**
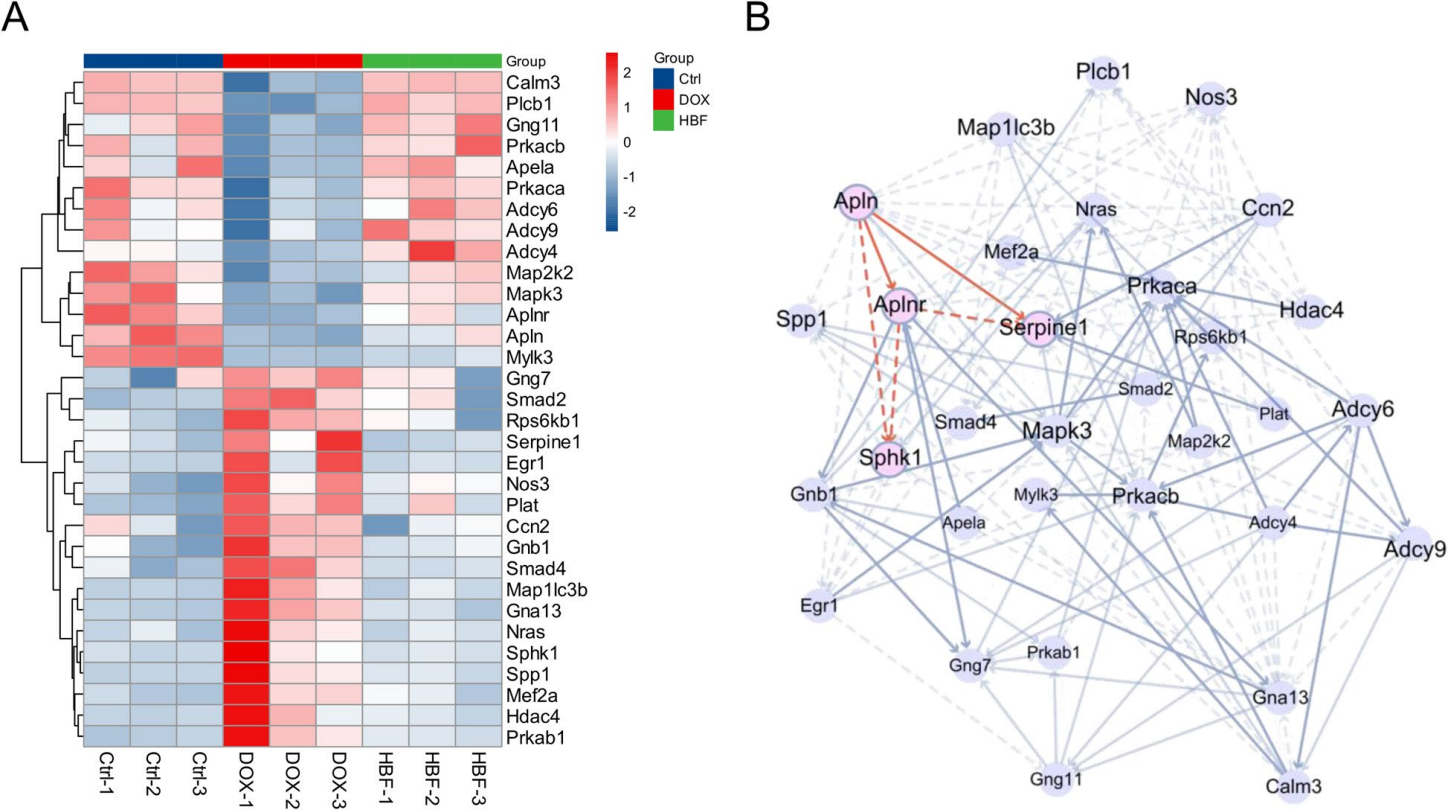
Exploring the molecular mechanisms through which HBF exerts its regulatory effects on pathological processes. **A** Heatmap of DEGs in the apelin signaling pathway. **B** PPI networks of DEGs within the apelin signaling pathway

To sort out the molecular relationships by which the Apelin signaling pathway is regulated, we analysed the upstream and downstream relationships of the reverse-regulated genes in the pathway. Within the PPI network of the reverse-regulated genes, Apln was located at the most upstream position. In conjunction with text mining, we focused on two downstream genes: Sphk1 and Serpine1 (Fig. 3A, B). Sphk1 is an ATP-dependent lipid kinase that is implicated in the pathogenesis of various diseases and has emerged as a promising therapeutic target for inflammatory conditions because of its intracellular overexpression. Studies have demonstrated that DOX can induce extensive oxidative stress, with the resultant ROS suppressing the production of S1P, thereby weakening S1P’s inhibitory effect on SphK1, leading to elevated expression levels of SphK1 [39, 40]. The enhanced activity of SphK1 can further induce the phosphorylation of NF-κB p65, triggering an inflammatory response, while the increased levels of apelin can inhibit this process [41–43]. Serpine1, another downstream gene of the apelin signaling pathway, encodes the PAI-1, an inhibitor of the endogenous fibrinolytic system. The expression levels of PAI-1 are influenced by factors such as ROS, inflammatory cytokines, TGF-β, and epidermal growth factor (EGF) [44]. Notably, ROS are considered initiators of fibrosis; when oxidative stress occurs, the levels of ROS increase, activating TGF-β, which leads to the phosphorylation of SMAD2/3 and the initiation of PAI-1 transcription. Due to the positive feedback loop of regulation between PAI-1 and TGF-β, this leads to the formation of extracellular matrix (ECM) [45–48]. Therefore, our study focused on two signaling axes and their associated pathological processes: oxidative stress and the immune-inflammatory response mediated by the Apelin/SphK1/NF-κB signaling axis and the pathological processes associated with ECM formation mediated by Apelin/PAI-1/TGFβ.

### HBF regulates the Apelin/SphK1/NF-κB and Apelin/PAI-1/TGFβ signaling axis and dependent pathological processes

The results of the RNA-Seq analysis revealed two key findings: first, HBF may have a regulatory effect on the Apelin/SphK1/NF-κB signaling axis, influencing oxidative stress and the immunoinflammatory response; second, HBF may regulate the Apelin/PAI-1/TGFβ signaling axis, affecting ECM formation. We subsequently performed experimental validation.

**Fig. 4:**
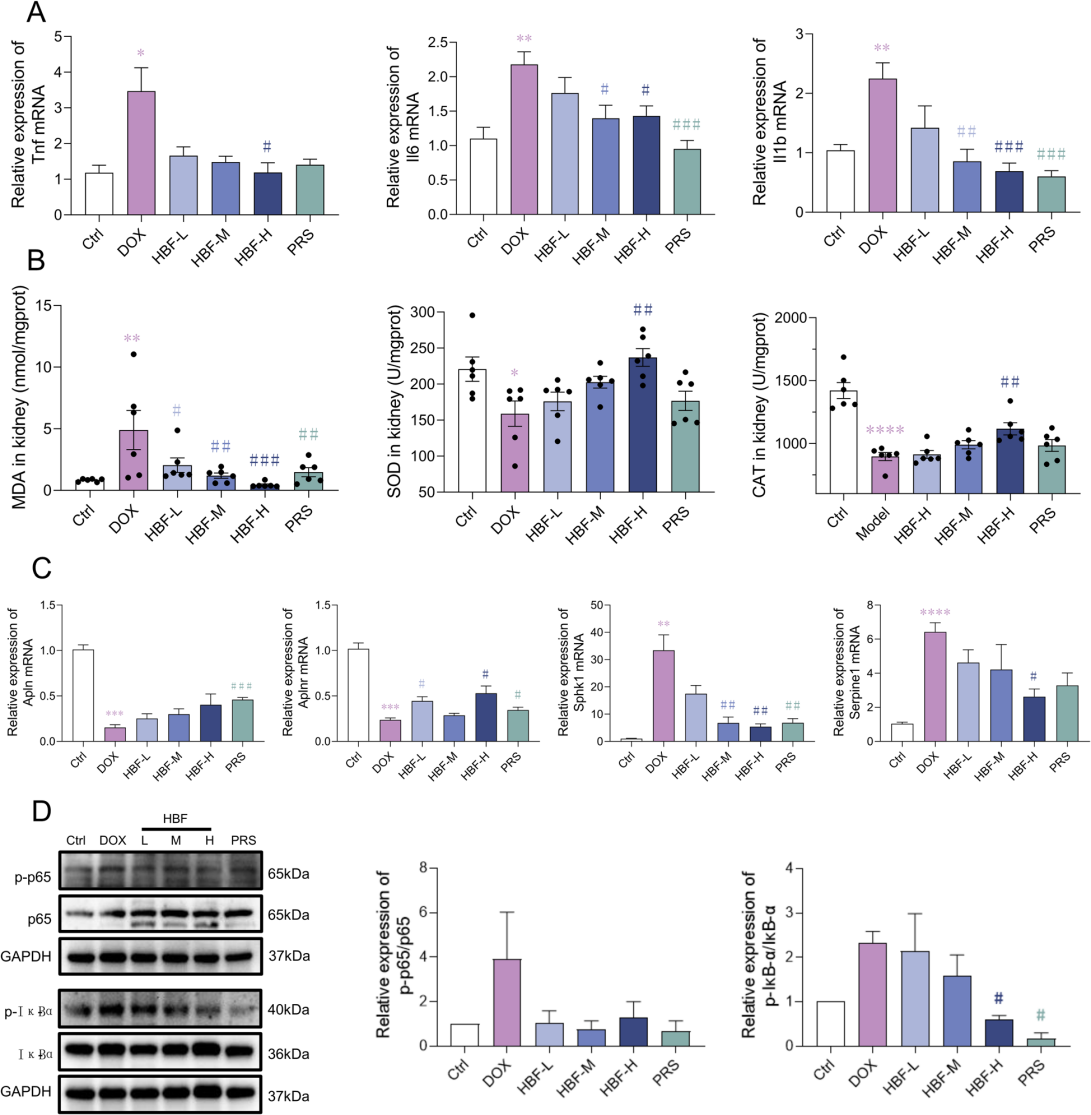
Examination of HBF's role in the immune-inflammatory response, oxidative stress, and corresponding renal signaling axis. **A** mRNA expression levels of three inflammatory factors in renal tissues; n = 3 per group. **B** Levels of the oxidative stress product MDA and the antioxidant enzymes SOD and CAT in renal tissues; n = 6 per group. **C** mRNA expression levels of important targets of the Apelin/SphK1/NF-κB and Apelin/PAI-1/TGFβ signaling axes; n = 3 per group. **D** Expression of important targets of the Apelin/SphK1/NF-κB signaling axis, as detected by Western blotting; n = 3 per group. (Data are presented as the mean ± SEM, *p < 0.05 vs. Ctrl; **p < 0.01 vs. Ctrl; ***p < 0.001 vs. Ctrl; ****p < 0.0001 vs. Ctrl, #*p* < 0.05 vs. DOX, ##*p* < 0.01 vs. DOX; ###p < 0.001 vs. DOX)

The expression levels of inflammatory factors in mouse kidney tissues were assessed using qPCR, revealing that HBF administration significantly suppressed their expression, which to some extent confirmed the modulation of immune-inflammatory responses in kidney tissues by HBF (Fig. 4A). In terms of oxidative stress, HBF administration significantly suppressed the increase in the level of MDA, a lipid peroxidation product induced by DOX, and restored the levels of the antioxidant enzymes SOD and CAT (Fig. 4B). This result also supports the role of HBF in the regulation of oxidative stress in renal tissues. Analysis of the Apelin/SphK1/NF-κB signaling axis, which mediates immune-inflammatory responses and oxidative stress, revealed that the expression levels of Apln and apelin receptor (Aplnr) in the HBF-treated group were greater than those in the DOX group, whereas the expression levels of Sphk1 and Serpine1 were lower (Fig. 4C). Western blotting revealed that the phosphorylation levels of IκB-α and NF-κB downstream of Sphk1 were reduced after HBF administration (Fig. 4D).

**Fig. 5:**
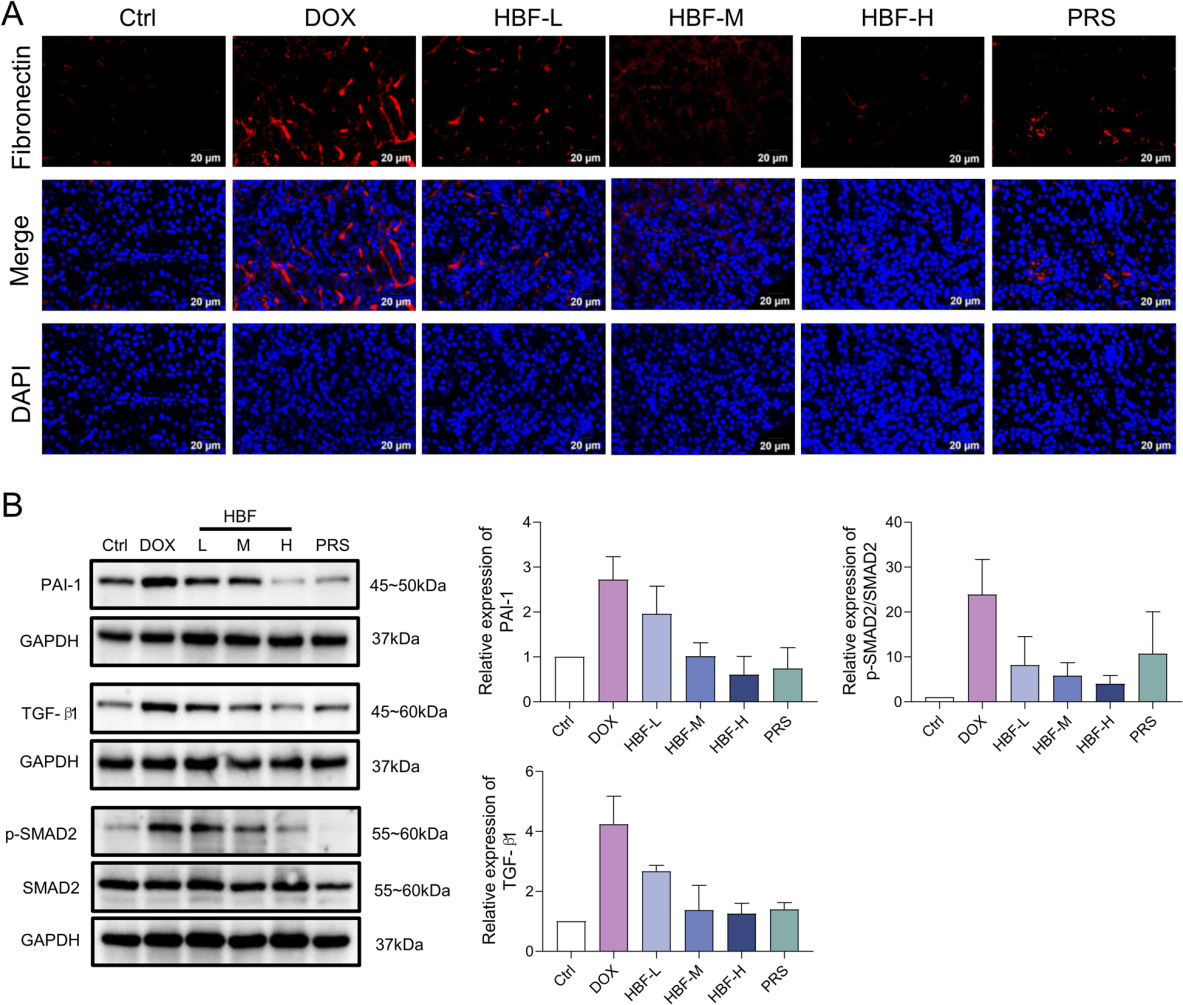
Examination of HBF’s role in ECM formation and corresponding renal signaling axis. **A** IF staining results for fibronectin in renal tissue sections. **B** Expression of important targets of the Apelin/PAI-1/TGFβ signaling axis, as detected by Western blotting; n = 3 per group. The data are presented as the means ± SEMs

Fibronectin is a marker of the ECM, and positive fluorescent signals were predominantly localized in the renal tubular mesenchyme, and the fluorescence intensity was stronger in the DOX group than in the HBF group (Fig. 5A). These findings indicate that HBF has an inhibitory effect on this pathological process of ECM formation. Evaluation of the Apelin/PAI-1/TGFβ signaling axis, which mediates ECM formation, revealed lower expression levels of PAI-1 and TGF-β1, as well as lower phosphorylation levels of mothers against decapentaplegic homologue 2 (SMAD2), in the HBF-treated group than in the DOX-treated group (Fig. 5B).

The Apelin signaling pathway has been extensively studied for its role in renal physiology and disease. It is considered a potential target for the treatment of various kidney diseases due to its involvement in regulating renal hemodynamics, promoting diuresis, and its protective effects in a variety of kidney injury models [49]. These findings highlight the role of HBF in the three pathological processes of the immune-inflammatory response, oxidative stress, and ECM formation in renal tissues, as well as the modulation of two signaling axes: the Apelin/SphK1/NF-κB axis and the Apelin/PAI-1/TGFβ axis.

### Screening of SphK1 inhibitors in HBF

The results from the above experiments indicate that SphK1 serves as a critical mediator of the immune-inflammatory response and oxidative stress. To investigate the potential active ingredients within HBF that influence these advantageous pathological processes, we screened HBF inhibitors that target SphK1.

**Fig. 6:**
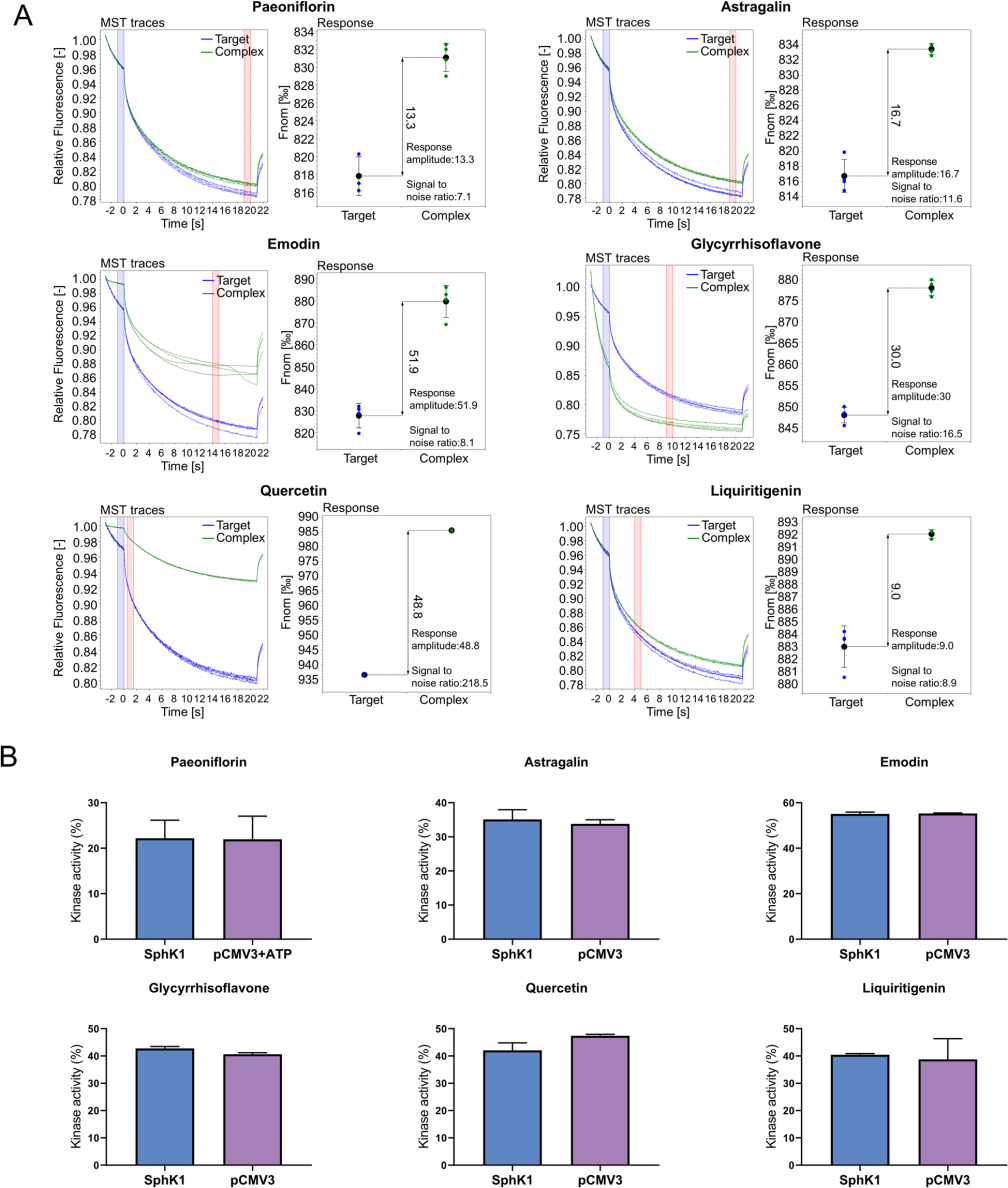
Six compounds in HBF inhibit the activity of SphK1 by directly binding to it. **A** MST traces and signal-to-noise ratio for compounds that interact with GFP-SphK1 at a concentration of 200 μM. Paeoniflorin, astragalin, emodin, glycyrrhisoflavone, quercetin, and liquiritigenin exhibit affinity potential for GFP-SphK1. **B** Six compounds that demonstrated affinity for SphK1 also inhibited its enzyme activity

In the process of screening for SphK1 inhibitors, we initially screened 28 prototype blood-entry compounds in HBF for their affinity potential by protein purification-free MST molecular interactions. For the binding assay, lysates from GFP-SphK1-overexpressing cells, which serve as binding targets, were subjected to quality assessment (Fig. S1A and B), and the signaling response of the binding assay was evaluated with PF-543, a known SphK1 inhibitor. The subsequent screening revealed that 12 out of the 28 compounds, including paeoniflorin, astragalin, emodin, glycyrrhisoflavone, quercetin, liquiritigenin, gallic acid, honokiol, ( +)-magnoflorine, rhein, licochalcone B, echinatin (Fig. 6A and S1C), demonstrated binding affinity to the target at a concentration of 200 μM.

To further evaluate these 12 compounds, we developed an ATP kinase activity assay using crude enzyme solutions. The inhibitory effects of these compounds on SphK1 were assessed by comparing their effects on the ATP-dependent kinase activities of both the control enzyme solution and the SphK1 enzyme solution. In the absence of the inhibitor, at the same cell lysate concentration, the ATP kinase activity of the SphK1 enzyme solution was significantly greater than that of the control enzyme solution, and when the SphK1 inhibitor inhibited the activity of all the SphK1 proteins in the enzyme solution, there was no difference between the ATP kinase activities of the SphK1 enzyme solution and the control enzyme solution.

The functionality of the assay system was validated by conducting a quality assessment through Western blotting and investigating the system’s response using PF-543 (Fig. S2A and B). Subsequent screening identified six compounds, namely, paeoniflorin, astragalin, emodin, glycyrrhisoflavone, quercetin, and liquiritigenin, as inhibitors of SphK1 (Fig. 6B).

### Screening of PAI-1 inhibitors in HBF

RNA-Seq analysis and experimental validation revealed that PAI-1 plays a pivotal role in mediating the pathological processes of ECM formation. To identify the ingredients in HBF that exhibit inhibitory effects on ECM formation, we conducted a screen to detect PAI-1 inhibitors among the prototype blood entry compounds of HBF.

**Fig. 7:**
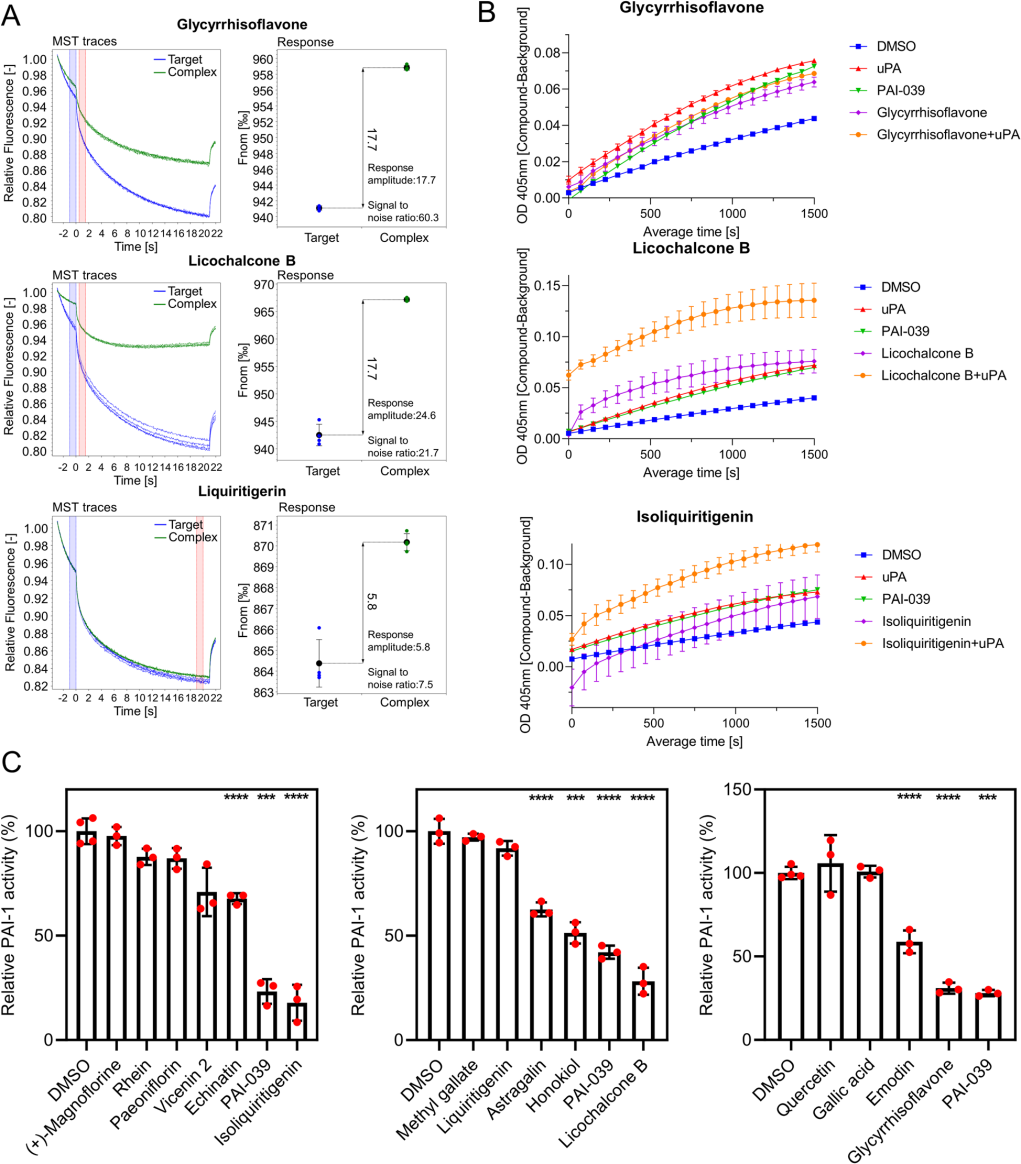
Three compounds in HBF inhibit the activity of PAI-1 by directly binding to it. **A** MST traces and signal‒to‒noise ratios for compounds that interact with GFP-PAI-1 at a concentration of 200 μM. Glycyrrhisoflavone, licochalcone B, and isoliquiritigenin exhibit affinity potential for GFP-PAI-1. **B** The dynamic curve of PAI-1 activity inhibited by 200 μM compound. Glycyrrhisoflavone, licochalcone B, and isoliquiritigenin inhibited PAI-1 activity by more than 50% at a concentration of 200 μM. **C** Effect of each compound on PAI-1 activity at 200 μM.

Similar to the SphK1 inhibitor screening process, we first constructed a protein purification-free MST molecular interaction system to screen for compounds with PAI-1 affinity potential from 28 prototype blood entry compounds of HBF. The quality of the GFP-PAI-1-overexpressing cell lysates used as interaction targets was validated through Western blotting, and the system’s ability to generate positive signals was confirmed with PAI-039, a known PAI-1 inhibitor. Subsequent screening revealed that at a concentration of 200 μM, 13 out of the 28 compounds displayed affinity for the target, including glycyrrhisoflavone, licochalcone B, liquiritigenin, paeoniflorin, quercetin, methyl gallate, vicenin 2, astragalin, isoliquiritigenin, echinatin, emodin, rhein, and ( +)-magnoflorine (Fig. 7A and Fig. S3C).

To assess the PAI-1 inhibitory activity of the 13 compounds, we utilized the purified PAI-1 protein to establish a uPA/PAI-1-catalysed substrate chromogenic system for detecting PAI-1 inhibitor activity (Figs. 7B and S4). The findings revealed that three compounds, glycyrrhisoflavone, licochalcone B, and isoliquiritigenin, decreased PAI-1 activity to less than 50% at a concentration of 200 μM (Fig. 7C).

## Discussion

To investigate the protective effect of HBF against AKI, our study used a DOX intraperitoneal injection-induced AKI model for pharmacological evaluation. According to the Foundation Kidney Disease Improving Global Outcomes (KDIGO) definition, AKI diagnosis involves a 1.5-fold increase in Cre compared with the baseline value within a week [50]. Compared with those in the Ctrl group, the Cre levels of the mice in the DOX group were threefold greater, indicating that the renal function of the model mice was severely impaired in a short period. Additionally, renal tubular vacuole-like degeneration, a hallmark of AKI, was prominently observed in the histopathological analysis. Consequently, the effective establishment of the AKI model was verified.

TCM identifies “dampness” and “heat” as pivotal factors contributing to kidney damage, leading to stasis known as “dampness and heat injure the kidneys” [51]. The concept emphasizes timely administration of “clearing heat and eliminating dampness” treatment for kidney protection [52]. Within HBF, each herb serves a distinct purpose in TCM: Huoxiang transforms dampness with its aroma, Cangzhu warms and dries dampness with bitterness, Fabanxia also warms and dries dampness, Fuling drains heat by transforming dampness, and Dahuang clears heat and dries dampness. Collectively, these herbs work together to alleviate renal injury caused by dampness and heat. Therefore, according to Chinese medicine theory, HBF is effective for prevention of AKI. In addition, modern medical research has shown that renal injury caused by dampness-heat is characterized by renal interstitial inflammatory cell infiltration and collagen deposition at the microscopic level, which aligns with the findings of the DOX-induced model in this study [51]. HBF has a therapeutic effect on these pathological features, which may be related to the effects of the herbs in this formula on dampness and heat.

Pathological processes encompass abnormal physiological functions and mechanisms in disease progression, involving a constellation of pathophysiological features. AKI is triggered by renal tubular epithelial cell necrosis and interstitial damage due to oxidative stress and an imbalanced immune-inflammatory response. Tubular atrophy follows, with collagenous deposits in the interstitium. Despite their diverse causes, AKIs share commonalities in pathological processes such as oxidative stress, immune‒inflammatory imbalance, cell death, and tissue remodelling [53–55]. This study was designed on the basis of these processes, characterizing indicators of each process in a pharmacological investigation of potential targets for HBF, including oxidative stress, immune and inflammatory responses, cell survival and death, and ECM formation. This study focused on identifying indicators in pharmacological studies of HBF targets, such as oxidative stress, immune responses, cell survival, and ECM formation. The results showed that HBF modulated various pathological components, demonstrating its wide range of effects. This finding suggests that targeting specific pathologies could effectively treat complex conditions such as AKI.

**Fig. 8:**
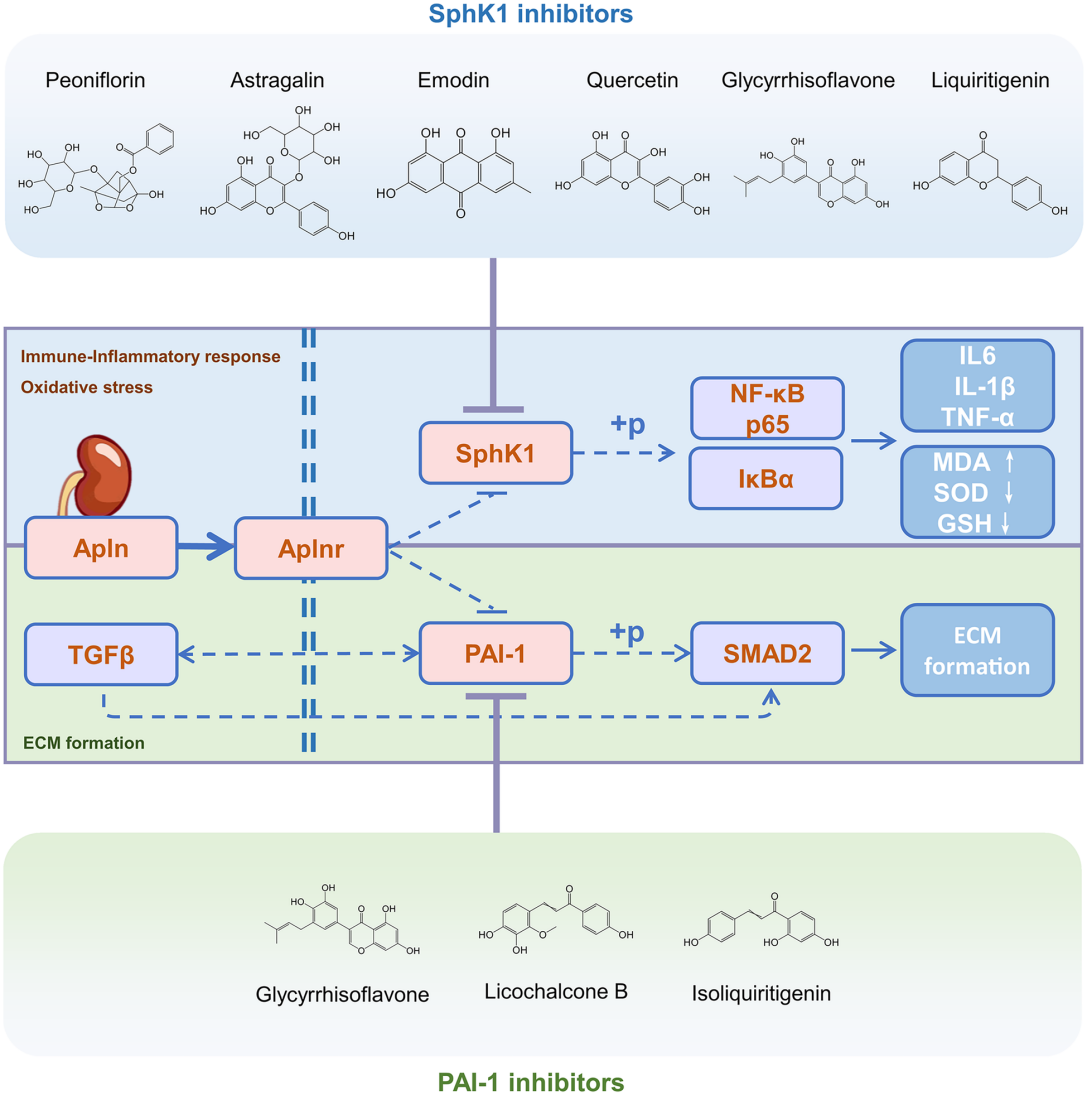
Molecular mechanisms by which HBF exerts a renoprotective effect. HBF can inhibit NF-κB phosphorylation and PAI-1/TGFβ signaling downstream by activating apelin signaling. This inhibition leads to decreased production of inflammatory factors, protection against ROS-induced damage, and a reduction in ECM formation, ultimately providing a protective effect against renal impairment

Through our exploration and validation, we have demonstrated that HBF affects AKI pathological processes through two apelin-mediated signaling axes, crucial endogenous signaling systems that are primarily distributed in the kidney [56]. These axes govern various downstream effects, including antioxidative stress, cell death, and ECM formation [57–59]. Activation of this system is believed to protect against AKI [37, 60–62]. One axis, the Apelin/SphK1/NF-κB axis, involves HBF-mediated inhibition of SphK1 expression and NF-κB phosphorylation via the activation of apelin, which ameliorates oxidative stress and immune-inflammatory responses. The other axis, Apelin/PAI-1/TGFβ, involves the upregulation of PAI-1 and TGFβ expression in HBF, hindering ECM formation through the positive feedback loop between PAI-1 and TGFβ (Fig. 8). SphK1, which is widely distributed in renal tissues and pivotal for downstream regulation, is a potential target for renal disease treatment [63, 64]. Elevated PAI-1 levels during inflammation impede the fibrinolytic system, making PAI-1 inhibition a novel antirenal fibrosis drug development strategy [65]. Therefore, our screening focused on active ingredients targeting SphK1 and PAI-1. Two inhibitor screening strategies were used for each target: first, compounds with potential target affinity were screened using protein purification-free MST, followed by further screening of compounds with target protein inhibitory activity via an enzyme-catalysed reaction system. The protein purification-free MST molecular intercalation method, which uses a GFP fluorescent marker fused to the protein for marking and cell lysates for detection, provides an easy and uncomplicated alternative to traditional protein purification-requiring methods [66]. For further screening, we used SphK1 ATP-dependent enzyme catalysis and PAI-1 uPA enzyme activity inhibition. However, screening effective compounds surrounding these targets encounters challenges in elucidating the pharmacological substance basis. Therefore, future investigations should expand the screening scope and focus on establishing quantitative-effect relationships for these active ingredients.

## Conclusion

In this study, the protective effect of HBF on renal function was validated through animal experiments. The mechanism of these effects was investigated and confirmed to be associated primarily with Apelin/SphK1/NF-κB-mediated oxidative stress and immune-inflammatory responses, as well as with Apelin/PAI-1/TGFβ-mediated ECM formation. Six inhibitors targeting SphK1 were identified, namely, paeoniflorin, astragalin, emodin, glycyrrhisoflavone, quercetin, and liquiritigenin. Additionally, three inhibitors were discovered for PAI-1, including glycyrrhisoflavone, licochalcone B, and isoliquiritigenin. These inhibitors exert direct affinity inhibition on their respective targets.

## Supplementary Information

Supplementary material 1: Table S1. GO-BP source terms for the pathological process gene sets. Table S2. Combined FC values for PathExNET analysis of the shared signaling pathways associated with the four pathological processes. Table S3. Primer sequences for RT‒PCR analysis. Table S4. List of compounds in HBF to be screened. Table S5. Groups of the uPA/PAI-1 catalysed substrate chromogenic reaction group setting.

Supplementary material 2: Fig. S1. Protein-free purification of SphK1 by MST for cell lysate quality control, affinity testing, and screening of compounds for affinity potential. Fig. S2. Quality control and testing of the ATP-dependent kinase assay system and application to the screening of SphK1 inhibitors. Fig. S3. Protein-free purification of PAI-1 by MST for cell lysate quality control, affinity testing, and screening of compounds for affinity potential.

## Data Availability

The data in this study are available from the corresponding author upon reasonable request.

## Abbreviations

Alb: Albumin
BP: Biological processes
BUN: Urea nitrogen
CAT: Catalase
COVID-19: Corona Virus Disease 2019
Cre: Creatinine
DEGs: Differential gene analysis
DMEM: Dulbecco’s Modified Eagle Medium
DOX: Doxorubicin
ECM: Extracellular matrix
GFP: Green fluorescent protein
GMT: Gene matrix transposed
GO: Gene ontology
GSVA: Gene set variation analysis
HBF: Huashi Baidu formula
ICUs: Intensive Care Units
IL-1β: Interleukin-1 beta
IL-6: Interleukin-6
MDA: Malondialdehyde
MST: Microscale thermophoresis
PAI-1: Plasminogen activator inhibitor 1
PCA: Principal component analysis
pNA: P-Nitroaniline
PPI: Protein–protein interaction
PRS: Prednisone
S1P: Sphingosine 1-phosphate
SOD: Superoxide dismutase
SPF: Specific pathogen-free
Sph: Sphingosine
SphK1: Sphingosine kinase 1
TCM: Traditional Chinese Medicine
TNF-α: Tumor necrosis factor
TP: Total protein
uPA: Urokinase-type plasminogen activator
