# Supplementary material for: Protective effect of Huashi Baidu formula against AKI and active ingredients that target SphK1 and PAI-1
Source: Chin Med. 2024 Nov 1;19:152. doi: 10.1186/s13020-024-01024-7 (PMC11529477; doi:10.1186/s13020-024-01024-7)
Supplement: Supplementary file 1 — Supplementary material 1: Table S1. GO-BP source terms for the pathological process gene sets. Table S2. Combined FC values for PathExNET analysis of the shared signaling pathways associated with the four pathological processes. Table S3. Primer sequences for RT‒PCR analysis. Table S4. List of compounds in HBF to be screened. Table S5. Groups of the uPA/PAI-1 catalysed substrate chromogenic reaction group setting. [file 13020_2024_1024_MOESM1_ESM.docx]

**Supplementary Information**

***for***

**Protective effect of Huashi Baidu Formula against AKI and active ingredients that target SphK1 and PAI-1**

Yute Zhong ^a, b^, Xia Du ^b, c^, Ping Wang ^b^, Weijie Li ^b^, Cong Xia ^b^, Dan Wu ^b^, Hong Jiang ^b^, Haiyu Xu ^b, d*^ & Luqi Huang^e*^

^a^ College of Chinese Medicinal Materials, Jilin Agricultural University, Changchun 130118, Jilin, China

^b^ Institute of Chinese Materia Medica, China Academy of Chinese Medical Sciences, Beijing 100700, China

^c^ Institute of Traditional Chinese Medicine, Shaanxi Academy of Traditional Chinese Medicine, Xi'an, China

^d^ State Key Laboratory for Quality Ensurance and Sustainable Use of Dao-di Herbs，Institute of Chinese Materia Medica,China Academy of Chinese Medical Sciences, 100700

^e^ National Resource Center for Chinese Materia Medica, China Academy of Chinese Medical Sciences, Beijing, 100700, China.

^*^ Correspondence to Luqi Huang and Haiyu Xu

# Supplementary Table S1. GO-BP source terms for pathological aspect gene sets.

| **Pathological aspects** | **GO-BP terms（ID）** |
| --- | --- |
| Oxidative stress | GO:0034599, GO:0008631, GO:1900408, GO:1902176, GO:1903377, GO:1902883, GO:0036480, GO:1902177, GO:1900407, GO:1902175, GO:1902882, GO:0043619, GO:0006979 |
| Immune response | GO:0002253, GO:0002218, GO:0002250, GO:0002460, GO:0140367, GO:0061760, GO:0061844, GO:0140374, GO:0002312, GO:0002322, GO:0002263, GO:0002367, GO:0006959, GO:0002455, GO:0002767, GO:0002765, GO:0002768, GO:0002433, GO:0002764, GO:0002418, GO:0002381, GO:0045087, GO:0002220, GO:0002758, GO:0002227, GO:0002285, GO:0002281, GO:0002279, GO:0002313, GO:0002275, GO:0002323, GO:0002423, GO:0002820, GO:0002719, GO:0002921, GO:0002924, GO:0050777, GO:0045824, GO:0033007, GO:0002701, GO:0002829, GO:2000317, GO:0002826, GO:0002283, GO:0002251, GO:0002821, GO:0002720, GO:0002922, GO:0002925, GO:0050778, GO:0033008, GO:0061081, GO:0002857, GO:0002702, GO:0002830, GO:0002842, GO:2000318, GO:0002827, GO:0002440, GO:0002819, GO:1905034, GO:0002920, GO:0002923, GO:0050776, GO:0045088, GO:0033006, GO:0032826, GO:0002855, GO:0002700, GO:0002828, GO:2000316, GO:0002825, GO:0002208, GO:0002461, GO:0042092, GO:0002286, GO:0002292, GO:0002424, GO:0002309, GO:0072538, GO:0042088 |
| Inflammatory response | GO:0002526, GO:0002438, GO:0002544, GO:0002439, GO:0002248, GO:0002534, GO:0002349, GO:0006925, GO:0006954, GO:0002437, GO:0090594, GO:0002269, GO:0002232, GO:0002523, GO:0071608, GO:0002674, GO:0002865, GO:0002677, GO:1900016, GO:0050728, GO:0002862, GO:0002675, GO:0002866, GO:0002678, GO:1900017, GO:0050729, GO:0002863, GO:0002532, GO:0002673, GO:0002864, GO:0002676, GO:0050727, GO:0002861, GO:0106014, GO:0002536, GO:0002351, GO:0002246 |
| Cell death and survival | GO:0006921, GO:0097194, GO:1900118, GO:1900119, GO:2001270, GO:1900117, GO:0000422, GO:0030242, GO:0061684, GO:0016237, GO:0016236, GO:0010507, GO:1903147, GO:0016242, GO:0034727, GO:0010508, GO:1903599, GO:0016239, GO:0071211, GO:0010506, GO:1903146, GO:1904923, GO:0016241, GO:0061912, GO:0097707, GO:0140894, GO:0007042, GO:0097212, GO:0016237, GO:0007041, GO:0035751, GO:0033299, GO:0051315, GO:0040001, GO:0000278, GO:0007093, GO:0045448, GO:0098763, GO:0044772, GO:1903047, GO:0007076, GO:0000281, GO:1902410, GO:0044774, GO:1902969, GO:0033314, GO:0044819, GO:0007095, GO:0044818, GO:0031573, GO:0007080, GO:0140014, GO:0101024, GO:0006312, GO:0007064, GO:0000070, GO:0051306, GO:0090307, GO:0000022, GO:0007052, GO:0045930, GO:1901991, GO:0045950, GO:1900087, GO:0045931, GO:1901992, GO:0090267, GO:1903490, GO:0045840, GO:0062033, GO:1901970, GO:1902423, GO:0007346, GO:1901990, GO:0046602, GO:1902412, GO:0007088, GO:0000019, GO:0033047, GO:1901673, GO:0000083, GO:0000320, GO:1901563, GO:0140639, GO:0070269 |
| ECM formation | GO:1990000, GO:0072378, GO:0030199, GO:0090674, GO:0042730, GO:0072537, GO:0044346, GO:0090269, GO:0008543, GO:0010761, GO:0048144, GO:0036446, GO:1905907, GO:0051918, GO:2000270, GO:0090272, GO:0040037, GO:0010764, GO:0048147, GO:1904906, GO:2000271, GO:0090271, GO:0045743, GO:0010763, GO:0048146, GO:0090080, GO:1905906, GO:1904847, GO:1904026, GO:0051917, GO:2000269, GO:0040036, GO:0010762, GO:0048145, GO:1904760, GO:0071774 |

# Supplementary Table S2. Combine FC values for PathExNET analysis of shared signalling pathways in four pathological aspects.

| **Pathway** | **DOX_vs_Ctrl** | **DOX_vs_HBF** |
| --- | --- | --- |
| MAPK signaling pathway | 1.00 | 1.12 |
| Erbb signaling pathway | 0.97 | 0.98 |
| Ras signaling pathway | 1.03 | 0.98 |
| Rap1 signaling pathway | 0.97 | 1.01 |
| cAMP signaling pathway | 1.04 | 0.98 |
| Nf-κB signaling pathway | 0.82 | 1.18 |
| HIF-1 signaling pathway* | 1.02 | 1.10 |
| FoxO signaling pathway | 0.90 | 0.91 |
| Sphingolipid signaling pathway | 1.13 | 1.00 |
| Phospholipase d signaling pathway | 0.98 | 0.96 |
| PI3K-Akt signaling pathway | 0.86 | 1.02 |
| VEGF signaling pathway | 0.98 | 1.20 |
| Apelin signaling pathway* | 1.01 | 1.02 |
| JAK-STAT signaling pathway | 0.85 | 1.00 |
| TNF signaling pathway* | 1.06 | 1.08 |

# Supplementary Table S3. Primer sequences for RT-PCR analysis.

| **Primer** | **Sequence** |
| --- | --- |
| Sphk1 | Forward: 5’-GCTTCTGTGAACCACTATGCTGG-3’  Reverse: 5’-ACTGAGCACAGAATAGAGCCGC-3’ |
| Serpine1 | Forward: 5’-GCCAGATTTATCATCAATGACTGGG-3’  Reverse: 5’-GGAGAGGTGCACATCTTTCTCAAAG-3’ |
| Tnf | Forward: 5’-TCTCATTCCTGCTTGTGGC-3’  Reverse: 5’-CACTTGGTGGTTTGCTACG-3’ |
| Il6 | Forward: 5’-CCGGAGAGGAGACTTCACAG-3’  Reverse: 5’-CATTTCCACGATTTCCCAGA-3’ |
| Il1b | Forward: 5’-AGCATCCAGCTTCAAATC-3’  Reverse: 5’-CTTCTCCACAGCCACAAT-3’ |
| Apln | Forward: 5’-TCTTGGCTCTTCCCTCTTTTCA-3’  Reverse: 5’-GTGCTGGAATCCACTGGAGAA-3’ |
| Aplnr | Forward: 5’-TACGCCAGTGTCTTTTGCCT-3’ |
|  | Reverse: 5’-CACCATGACAGGCACAGCTA-3’ |
| Actb | Forward: 5’-TATGCTCTCCCTCACGCCATCC-3’  Reverse: 5’-GTCACGCACGATTTCCCTCTCAG-3’ |

# Supplementary Table S4. List of compounds in HBF to be screened

| **No.** | **Compound** | **CAS** |
| --- | --- | --- |
| 1 | 18β-Glycyrrhetinic acid | 471-53-4 |
| 2 | Glycyrrhizic acid | 1405-86-3 |
| 3 | Paeoniflorin | 23180-57-6 |
| 4 | Isoliquiritigenin | 961-29-5 |
| 5 | Gallic acid | 149-91-7 |
| 6 | Salicylic acid | 69-72-7 |
| 7 | Rhein | 478-43-3 |
| 8 | Honokiol | 35354-74-6 |
| 9 | Magnolol | 528-43-8 |
| 10 | Quercetin | 117-39-5 |
| 11 | Azelaic acid | 123-99-9 |
| 12 | Liquiritin | 551-15-5 |
| 13 | Liquiritigenin | 578-86-9 |
| 14 | Methyl gallate | 99-24-1 |
| 15 | (+)-Magnoflorine | 2141-09-5 |
| 16 | Echinatin | 34221-41-5 |
| 17 | Albiflorin | 39011-90-0 |
| 18 | Citric acid | 77-92-9 |
| 19 | Pogostone | 23800-56-8 |
| 20 | Licochalcone B | 58749-23-8 |
| 21 | Vicenin 2 | 23666-13-9 |
| 22 | Kynurenic acid | 13593-94-7 |
| 23 | Poricoic acid B | 137551-39-4 |
| 24 | Glycyrrhisoflavone | 116709-70-7 |
| 25 | Astragalin | 480-10-4 |
| 26 | Lactiflorin | 1361049-59-3 |
| 27 | Formononetin | 485-72-3 |
| 28 | Emodin | 518-82-1 |

# Supplementary Table S5. Groups of uPA/PAI-1 catalysed substrate chromogenic reaction group setting

| **Constituents** | **Test wells**  **(Compound)** | **Test positive control wells**  **(Compound-Ctrl)** | **Test background control wells**  **(Compound-Background)** | **Positive control wells**  **(POS-Ctrl)** | **Negative control wells**  **(NEG-Ctrl)** | **Background control well**  **(Background)** | **PAI-039 test wells**  **(PAI-039)** |
| --- | --- | --- | --- | --- | --- | --- | --- |
| Compound (1 μL) | Compound | Compound | Compound | DMSO | DMSO | DMSO | PAI-039 |
| PAI-1 working solution (39 μL) | PAI-1 | Buffer | Buffer | Buffer | PAI-1 | PAI-1 | PAI-1 |
| uPA working solution (5 μL) | uPA | uPA | Buffer | uPA | uPA | Buffer | uPA |
| S-2444 working solution (5 μL) | S-2444 | S-2444 | S-2444 | S-2444 | S-2444 | S-2444 | S-2444 |
