# Supplementary material for: Protective effect of Huashi Baidu formula against AKI and active ingredients that target SphK1 and PAI-1
Source: Chin Med. 2024 Nov 1;19:152. doi: 10.1186/s13020-024-01024-7 (PMC11529477; doi:10.1186/s13020-024-01024-7)
Supplement: Supplementary file 2 — Supplementary material 2: Fig. S1. Protein-free purification of SphK1 by MST for cell lysate quality control, affinity testing, and screening of compounds for affinity potential. Fig. S2. Quality control and testing of the ATP-dependent kinase assay system and application to the screening of SphK1 inhibitors. Fig. S3. Protein-free purification of PAI-1 by MST for cell lysate quality control, affinity testing, and screening of compounds for affinity potential. [file 13020_2024_1024_MOESM2_ESM.docx]

**Supplementary Information**

***for***

**Protective effect of Huashi Baidu Formula against AKI and active ingredients that target SphK1 and PAI-1**

Yute Zhong ^a, b^, Xia Du ^b, c^, Ping Wang ^b^, Weijie Li ^b^, Cong Xia ^b^, Dan Wu ^b^, Hong Jiang ^b^, Haiyu Xu ^b, d*^ & Luqi Huang^e*^

^a^ College of Chinese Medicinal Materials, Jilin Agricultural University, Changchun 130118, Jilin, China

^b^ Institute of Chinese Materia Medica, China Academy of Chinese Medical Sciences, Beijing 100700, China

^c^ Institute of Traditional Chinese Medicine, Shaanxi Academy of Traditional Chinese Medicine, Xi'an, China

^d^ State Key Laboratory for Quality Ensurance and Sustainable Use of Dao-di Herbs，Institute of Chinese Materia Medica,China Academy of Chinese Medical Sciences, 100700

^e^ National Resource Center for Chinese Materia Medica, China Academy of Chinese Medical Sciences, Beijing, 100700, China.

^*^ Correspondence to Luqi Huang and Haiyu Xu


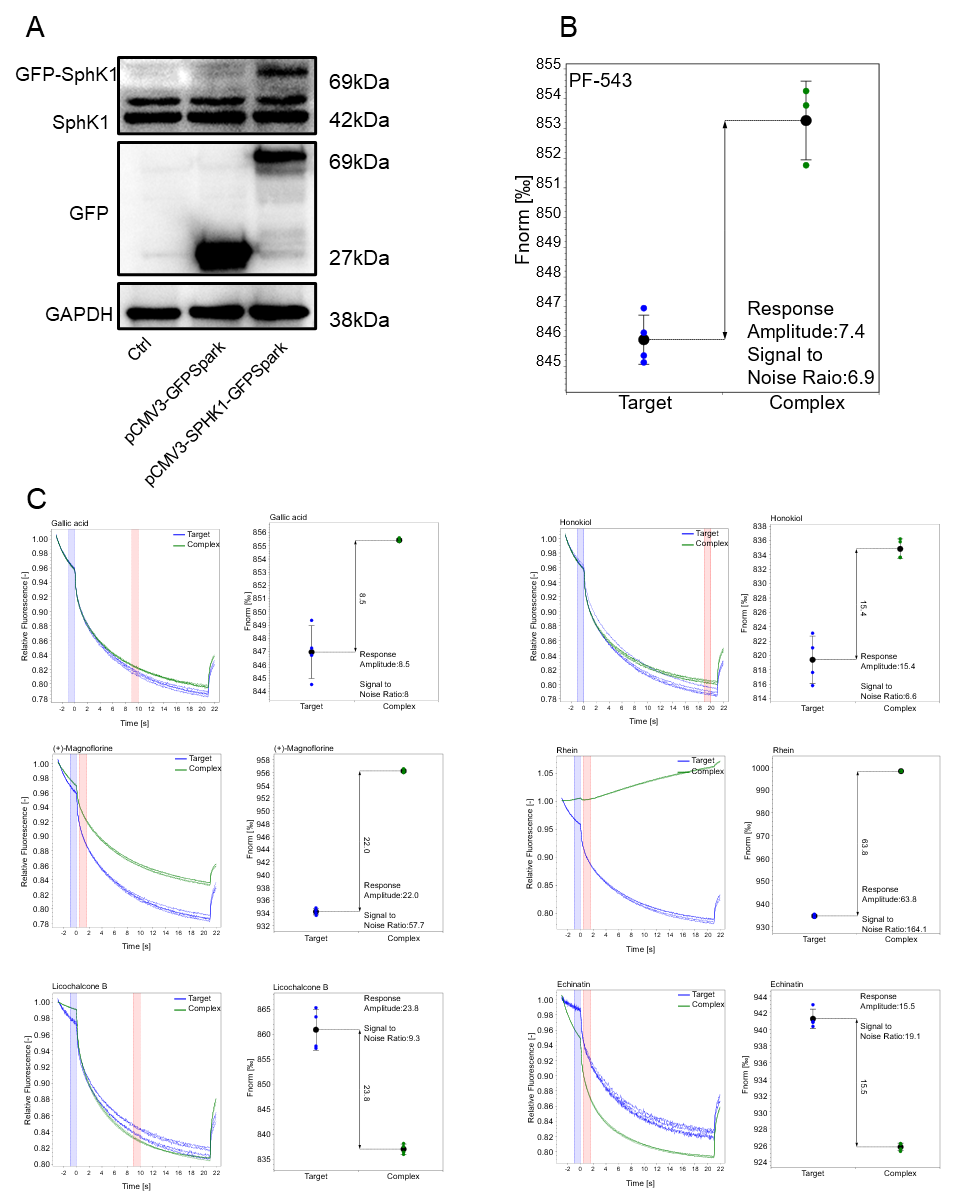


# Supplementary Fig. S1. Protein-free purification of SphK1 MST molecular interaction system for cell lysate quality control, affinity testing, and screening of compounds for affinity potential. (A) Western blot analysis was conducted to assess the quality of cell lysates. Lysates transfected with the pCMV3-GFPSpark plasmid showed a significant overexpression of GFP compared to control lysates, while lysates transfected with pCMV3-SPHK1-GFPSpark demonstrated a notable overexpression of the fusion protein GFP-SphK1. (B) Testing of the SphK1 MST molecular interaction system. Evaluation of the SphK1 inhibitor PF543 demonstrated a signal-to-noise ratio of 7.4, which was greater than 5.0, indicating a good signal-to-noise ratio. (C) The MST traces and signal-to-noise ratios of 6 out of 12 compounds exhibiting affinity potential with SphK1 partly by MST screening.


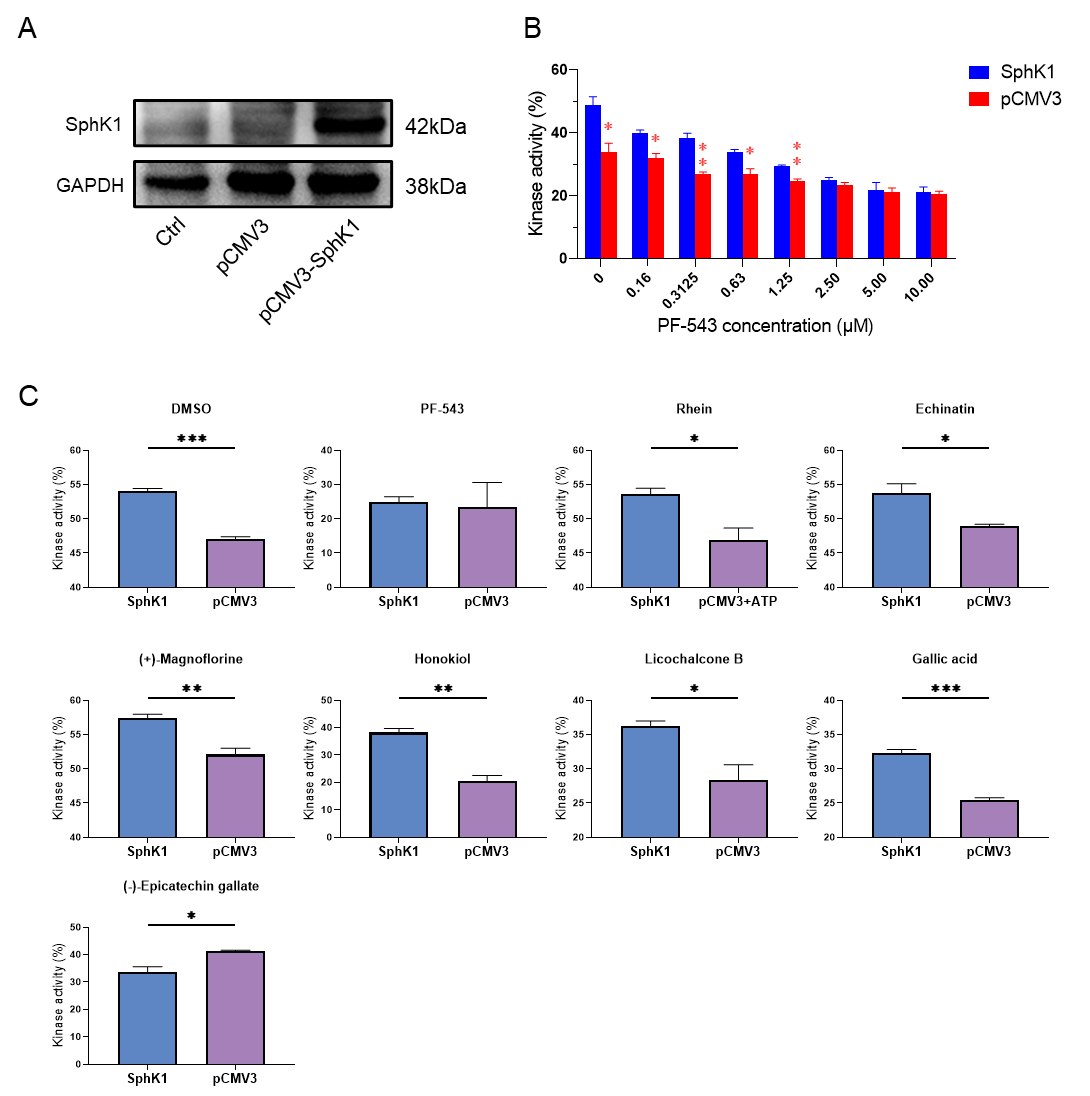


# Supplementary Fig. S2. Quality control and testing of the ATP-dependent kinase assay system, and application to the screening of SphK1 inhibitors. (A) Western blot analysis was conducted to assess the quality of cell lysates. Lysates transfected with the pCMV3 plasmid showed little to control lysates, while lysates transfected with pCMV3-SPHK1 demonstrated a notable overexpression of the SphK1. (B) The ATP-dependent kinase activity assay system was tested for its ability to respond to SphK1 inhibitors. With increasing concentrations of the SphK1 inhibitor PF543, SphK1 activity in the system was progressively inhibited, reaching complete inhibition above a specific threshold. At this time, there was no significant difference in ATP consumption between the pCMV3 control enzyme solution and the SphK1 crude enzyme solution, indicating consistent ATP-dependent kinase activity between the two enzyme solutions. The test results indicated that SphK1 activity in both the pCMV3 control enzyme solution and the SphK1 crude enzyme solution was entirely inhibited when the concentration of PF543 exceeded 2.5 μM, leading to similar levels of ATP-dependent kinase activity in both enzyme solutions. (C) Effect of compounds at 200 μM concentration on the ATP consumption capacity of two crude enzyme solutions. Despite exhibiting an affinity potential for SphK1, the seven compounds shown in the figure did not inhibit SphK1 activity.


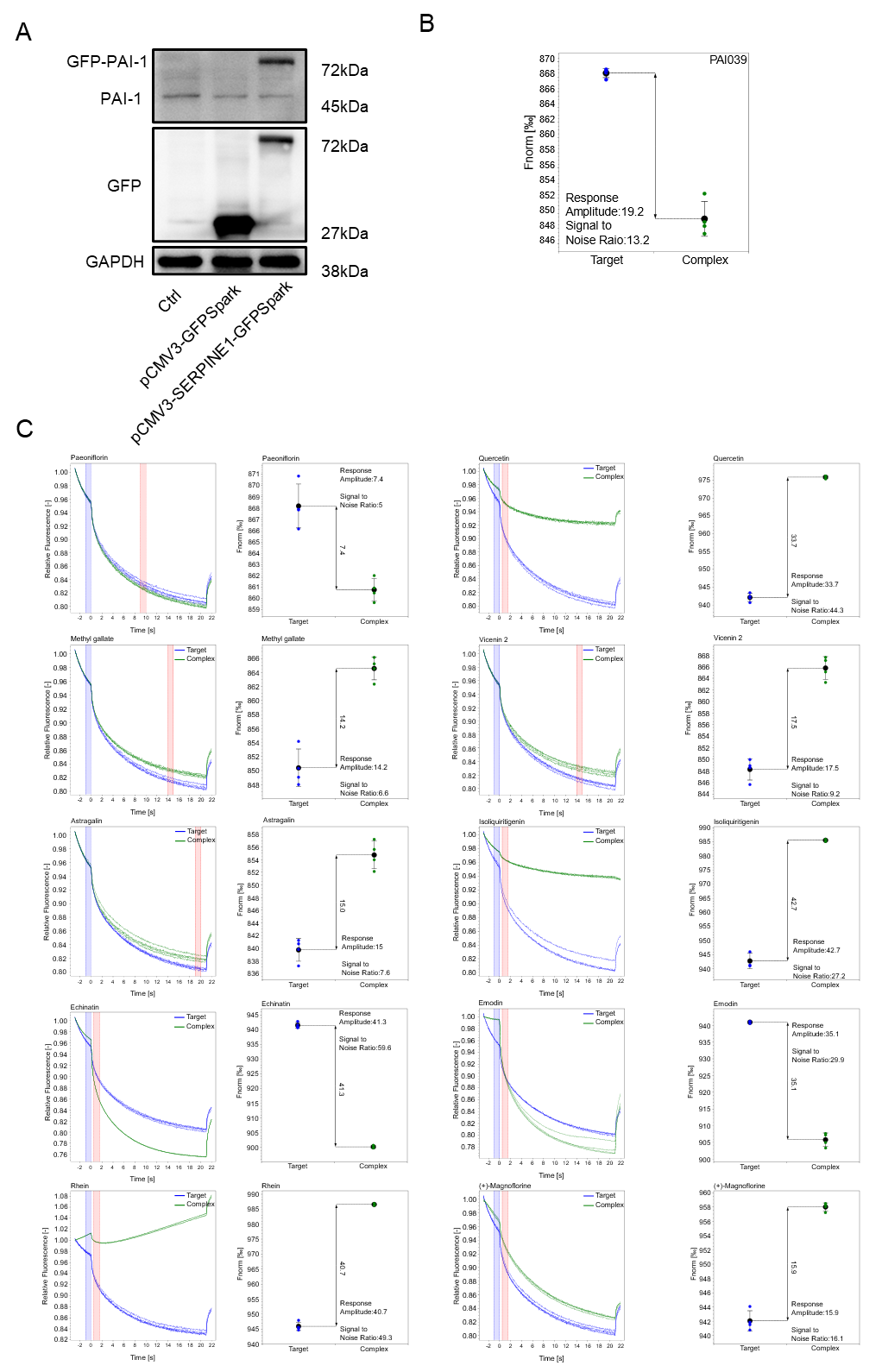


# Supplementary Fig. S3. Protein-free purification of PAI-1 MST molecular interaction system for cell lysate quality control, affinity testing, and screening of compounds for affinity potential. (A) Western blot analysis was conducted to assess the quality of cell lysates. Lysates transfected with the pCMV3-GFPSpark plasmid showed a significant overexpression of GFP compared to control lysates, while lysates transfected with pCMV3-SERPINE1-GFPSpark demonstrated a notable overexpression of the fusion protein GFP-PAI-1. (B) Testing of the PAI-1 MST molecular interaction system. Evaluation of the PAI-1 inhibitor PAI-039 demonstrated a signal-to-noise ratio of 13.2, which was greater than 5.0, indicating a good signal-to-noise ratio. (C) The MST traces and signal-to-noise ratios of 10 out of 13 compounds exhibiting affinity potential with PAI-1 partly by MST screening.


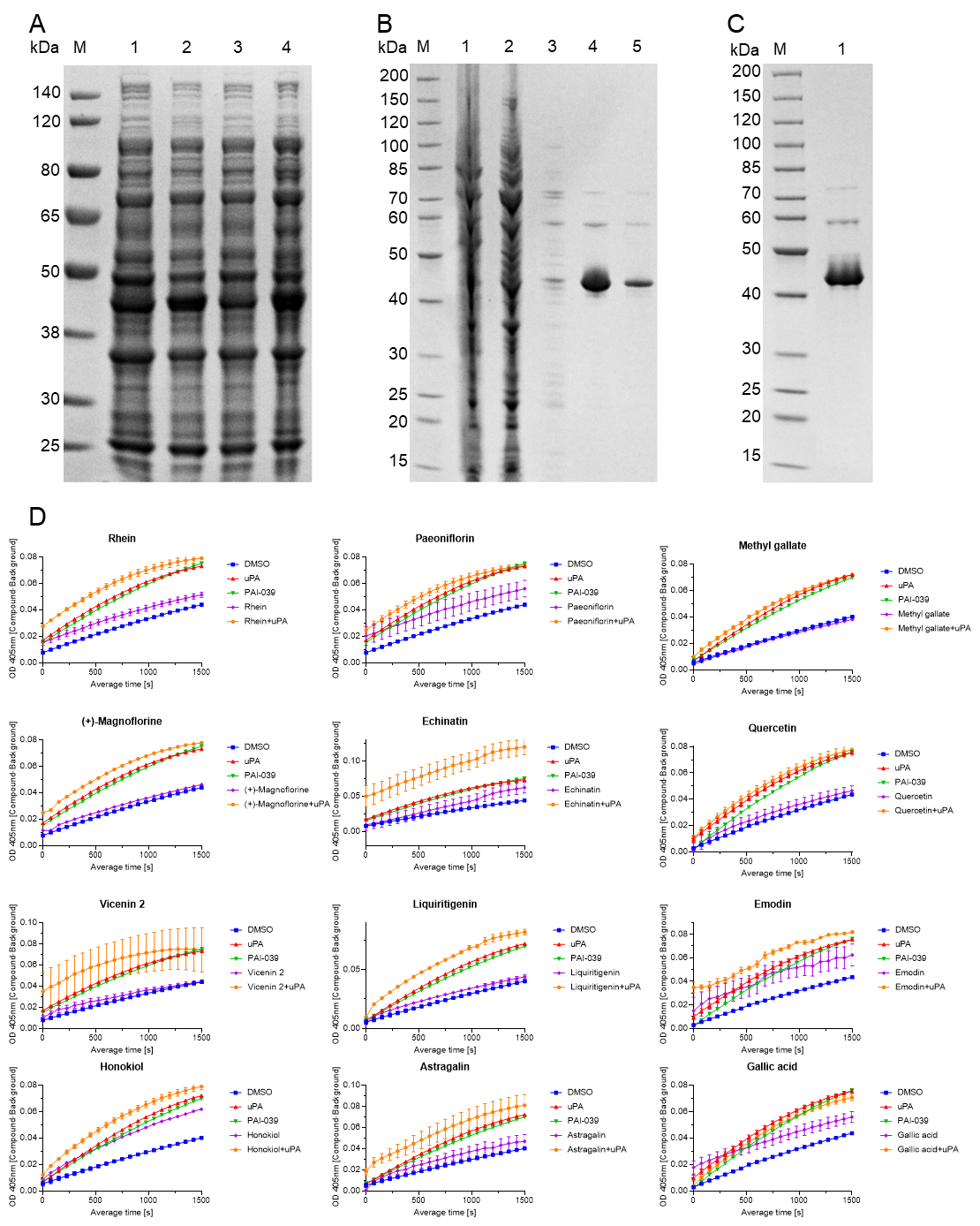


# Supplementary Fig. S4. The dynamic curve of PAI-1 activity inhibited by 200 μM compounds. The compounds shown were unable to reduce the activity of PAI-1 to less than 50% at a concentration of 200 μM.
